# Na_v_1.7 as a chondrocyte regulator and therapeutic target for osteoarthritis

**DOI:** 10.1038/s41586-023-06888-7

**Published:** 2024-01-03

**Authors:** Wenyu Fu, Dmytro Vasylyev, Yufei Bi, Mingshuang Zhang, Guodong Sun, Asya Khleborodova, Guiwu Huang, Libo Zhao, Renpeng Zhou, Yonggang Li, Shujun Liu, Xianyi Cai, Wenjun He, Min Cui, Xiangli Zhao, Aubryanna Hettinghouse, Julia Good, Ellen Kim, Eric Strauss, Philipp Leucht, Ran Schwarzkopf, Edward X. Guo, Jonathan Samuels, Wenhuo Hu, Mukundan Attur, Stephen G. Waxman, Chuan-ju Liu

**Affiliations:** 1 Department of Orthopaedic Surgery, New York University Grossman School of Medicine, New York, NY USA; 2 Department of Orthopaedics and Rehabilitation, Yale University School of Medicine, New Haven, CT USA; 3 Department of Neurology, Yale School of Medicine, New Haven, CT USA; 4 Center for Neuroscience and Regeneration Research, Veterans Affairs Connecticut Healthcare, West Haven, CT USA; 5 Department of Biomedical Engineering, Columbia University, New York, NY USA; 6 Division of Rheumatology, Department of Medicine, New York University Grossman School of Medicine, New York, NY USA; 7 Human Oncology and Pathogenesis Program, Memorial Sloan Kettering Cancer Center, New York, NY USA; 8 Marie-Josée and Henry R. Kravis Center for Molecular Oncology, Memorial Sloan Kettering Cancer Center, New York, NY USA; 9 Department of Cell Biology, New York University Grossman School of Medicine, New York, NY USA

**Keywords:** Gene regulation, Osteoarthritis

## Abstract

Osteoarthritis (OA) is the most common joint disease. Currently there are no effective methods that simultaneously prevent joint degeneration and reduce pain^1^. Although limited evidence suggests the existence of voltage-gated sodium channels (VGSCs) in chondrocytes^2^, their expression and function in chondrocytes and in OA remain essentially unknown. Here we identify Na_v_1.7 as an OA-associated VGSC and demonstrate that human OA chondrocytes express functional Na_v_1.7 channels, with a density of 0.1 to 0.15 channels per µm^2^ and 350 to 525 channels per cell. Serial genetic ablation of Na_v_1.7 in multiple mouse models demonstrates that Na_v_1.7 expressed in dorsal root ganglia neurons is involved in pain, whereas Na_v_1.7 in chondrocytes regulates OA progression. Pharmacological blockade of Na_v_1.7 with selective or clinically used pan-Na_v_ channel blockers significantly ameliorates the progression of structural joint damage, and reduces OA pain behaviour. Mechanistically, Na_v_1.7 blockers regulate intracellular Ca^2+^ signalling and the chondrocyte secretome, which in turn affects chondrocyte biology and OA progression. Identification of Na_v_1.7 as a novel chondrocyte-expressed, OA-associated channel uncovers a dual target for the development of disease-modifying and non-opioid pain relief treatment for OA.

## Abstract (web summary)

The voltage-gated sodium channel Na_v_1.7 has a dual role in osteoarthritis—in chondrocytes, it promotes joint damage, and in dorsal root ganglia neurons, it increases pain transmission.

## Main

Osteoarthritis is a disabling, degenerative disorder distinguished by progressive joint failure^3^. Although it is currently unclear whether the primary cause of OA is cartilage damage, OA always involves cartilage breakdown and loss of the unique extracellular matrix that normally guarantees the compressive resilience essential for joint function^1^. OA chondrocytes undergo complex changes, including anabolic and catabolic alteration. Chondrocytes are central protagonists in this regulatory cascade—as the target of external biomechanical and biochemical stimuli, as well as the source of proteases, cytokines and mediators that regulate the deterioration of articular cartilage^4^. Despite the high prevalence and morbidity of OA, effective disease-modifying treatments are not currently available, and the molecular mechanisms involved in OA remain poorly understood.

Alongside significant loss of articular cartilage, the dominant clinical symptom of OA is pain^5^. Specialized peripheral sensory neurons are abundant in joint tissues, including synovium and subchondral bone^6^, and contribute to pain in OA. These neurons express unique repertoires of VGSCs^7^. There are nine distinct VGSCs (Na_v_1.1–Na_v_1.9), encoded by genes *SCN1A–SCN11A*^8^. Na_v_1.7, Na_v_1.8 and Na_v_1.9 are of particular interest as targets for pain treatment owing to their preferential expression in peripheral sensory neurons within dorsal root ganglia (DRG), and their roles in action potential initiation and propagation within peripheral pain pathways^7^. Modulation of DRG-expressed Na_v_1.8 can attenuate OA pain^9^. The critical role of Na_v_1.7 in pain signalling^10^ and genetic validation (severe pain with gain-of-function Na_v_1.7 mutations^11,12^ and insensitivity to pain with loss-of-function Na_v_1.7 mutations^13,14^) have further supported Na_v_1.7 as a therapeutic target for pain. Notably, Na_v_1.7 gain-of-function mutations increase pain sensitivity in some patients with OA^15^. A role of Na_v_1.7 in inflammatory pain is supported by observations in global Na_v_1.7 and DRG-specific knockout mice^16,17^. A role of DRG-expressed Na_v_1.7 in OA pain was supported by reduced OA pain following spinal administration of ProTx II, a Na_v_1.7-selective antagonist, in the monosodium iodoacetate (MIA)-induced model of OA^18^.

Although expression of VGSCs in excitable cells is well-known^8^, they have also been observed in cell types that are not considered electrically excitable, including astrocytes, microglia, macrophages and cancer cells^19^. Cartilage is avascular and aneural, but angiogenesis and sensory nerve growth into OA cartilage may contribute to OA pain^20^. In addition, the presence of tetrodotoxin (TTX)-sensitive VGSCs in rabbit chondrocytes has been reported^2^. Nevertheless, although there is evidence that the aberrant activation of VGSCs contributes to OA pain^21^, the presence and function of VGSC(s) in chondrocytes and their roles in OA progression and pain remain essentially unknown.

To identify novel, differentially expressed genes in OA, we performed RNA-sequencing (RNA-seq) analysis on normal and arthritic cartilage, and identified Na_v_1.7 as the only significantly upregulated OA-associated VGSC. Here we demonstrate that distinct from DRG-expressed Na_v_1.7, which is only involved in OA pain signalling, chondrocyte-expressed Na_v_1.7 regulates chondrocyte biology and OA progression. We demonstrate that Na_v_1.7 blockade protects joints from deterioration, and Na_v_1.7 blockade mediates its chondroprotective effects at least in part by regulating intracellular Ca^2+^ signalling and the chondrocyte secretome.

## Na_v_1.7 in chondrocytes, elevated in OA

**Extended Data Fig. 1:**
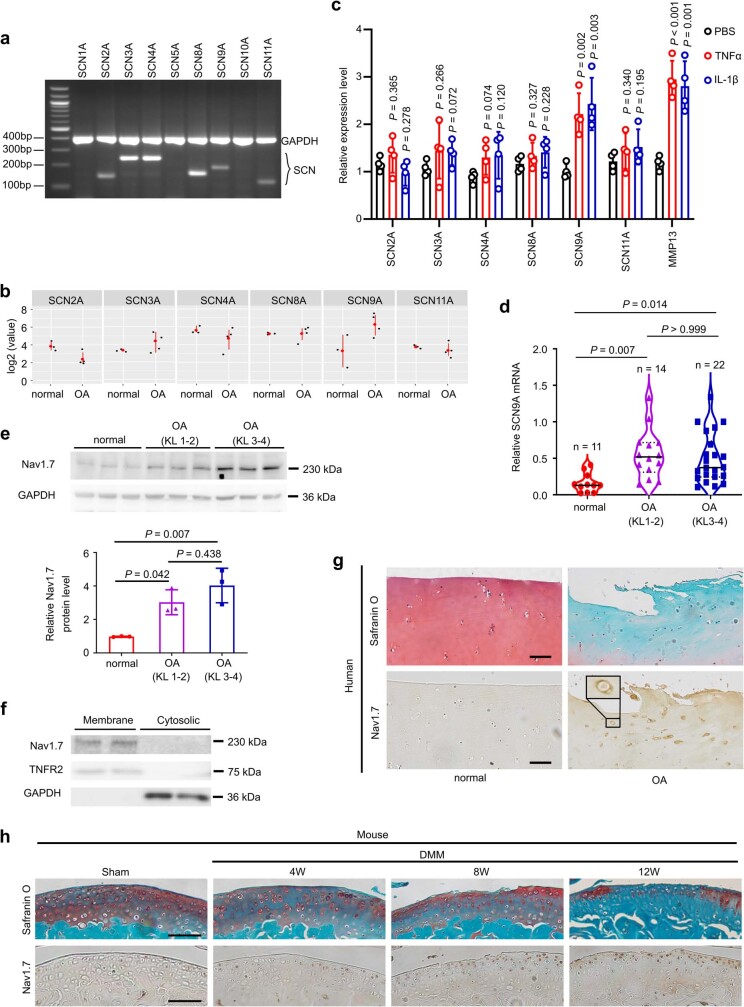
Nav1.7 is expressed in chondrocytes and upregulated in OA cartilage. **a**, Relative expression of 9 distinct VGSCs in human C28I2 chondrocytes, assayed by PCR. Expression of *GAPDH* mRNA serves as internal control. **b**, Relative expressions of VGSCs in human OA (n = 4) versus healthy (n = 3) cartilage, in RNA-sequencing data of human cartilage. **c**, The expression levels of chondrocytes expressed VGSCs in human C28I2 cells treated with 10 ng/ml TNFα or IL-1β for 24 h, assayed by qRT-PCR (n = 4 biological replicates). **d**, qPCR analysis of *SCN9A* in human cartilage from healthy individuals (n = 11) and from individuals with early stage (KL grade 1-2, n = 14) or late stage OA (KL grade 3-4, n = 22). **e**, Expression of Nav1.7 in cartilage from healthy individuals and OA patients with KL grade 1-2 or 3-4, assayed by Immunoblotting (n = 3 for each group). **f**, Membrane location of Nav1.7 in human chondrocytes detected by Immunoblotting (n = 3 biological replicates). **g**, Immunostaining of Nav1.7 in cartilage from healthy individuals and from individuals with late stage OA (n = 4 for each group). Scale bar = 100 µm. **h**, Immunostaining of Nav1.7 in cartilage collected from mice subjected to sham or DMM surgery (n = 4 mice per group). Scale bar = 100 µm. Data are means ± SD, *P* values are calculated by two-tailed unpaired Student’s t-test in **c** and one way ANOVA with Bonferroni post-hoc test in **d** and **e**. DMM, destabilization of medial meniscus; OA, osteoarthritis.

We assessed the expression profile of VGSCs in human chondrocytes using PCR with reverse transcription (RT–PCR) and found that, *SCN2A*, *SCN3A*, *SCN4A*, *SCN8A*, SCN*9A* and *SCN11A* are expressed in chondrocytes (Extended Data Fig. 1a). In line with this data, analysis of the genes that are differentially regulated between OA cartilage (Kellgren–Lawrence (KL) grade 3–4) and non-arthritic cartilage using our previous RNA-seq dataset^22^ (GSE168505) with a particular interest in VGSCs, indicated that six VGSCs were expressed in chondrocytes (Extended Data Fig. 1b). However, Na_v_1.7 mRNA (encoded by *SCN9A*) was the only VGSC transcript that was prominently upregulated (2.69 fold increased, *P* < 0.05) in OA cartilage compared with non-arthritic cartilage (Extended Data Fig. 1b). Among six VGSCs expressed in human chondrocytes, *SCN9A* expression was significantly induced by TNF and IL-1β, pro-inflammatory cytokines associated with OA^4^ (Extended Data Fig. 1c). Quantitative PCR with reverse transcription (RT–qPCR) analysis of Na_v_1.7 expression using mRNAs obtained from independent cartilage specimens (11 non-arthritic, 14 KL grade 1–2 OA and 22 KL grade 3–4 OA) confirmed that the Na_v_1.7 mRNA expression was significantly increased in both KL grade 1–2 and KL grade 3–4 OA cartilage compared with non-arthritic cartilage (Extended Data Fig. 1d). Na_v_1.7 protein level was also increased in all grades of radiographic severity (KL 1–4) of human OA compared with non-arthritic controls (Extended Data Fig. 1e). Membrane localization of Na_v_1.7 in chondrocytes was confirmed following fractionation of human chondrocytes and western blotting by using cytosolic and membrane fractions (Extended Data Fig. 1f). Immunohistochemical staining demonstrated increased expression of Na_v_1.7 in both human and mouse OA cartilage (Extended Data Fig. 1g,h). Collectively, these data demonstrate that Na_v_1.7 is expressed in chondrocytes and associates with OA progression.

## OA chondrocyte Na_v_1.7 electrophysiology

**Fig. 1:**
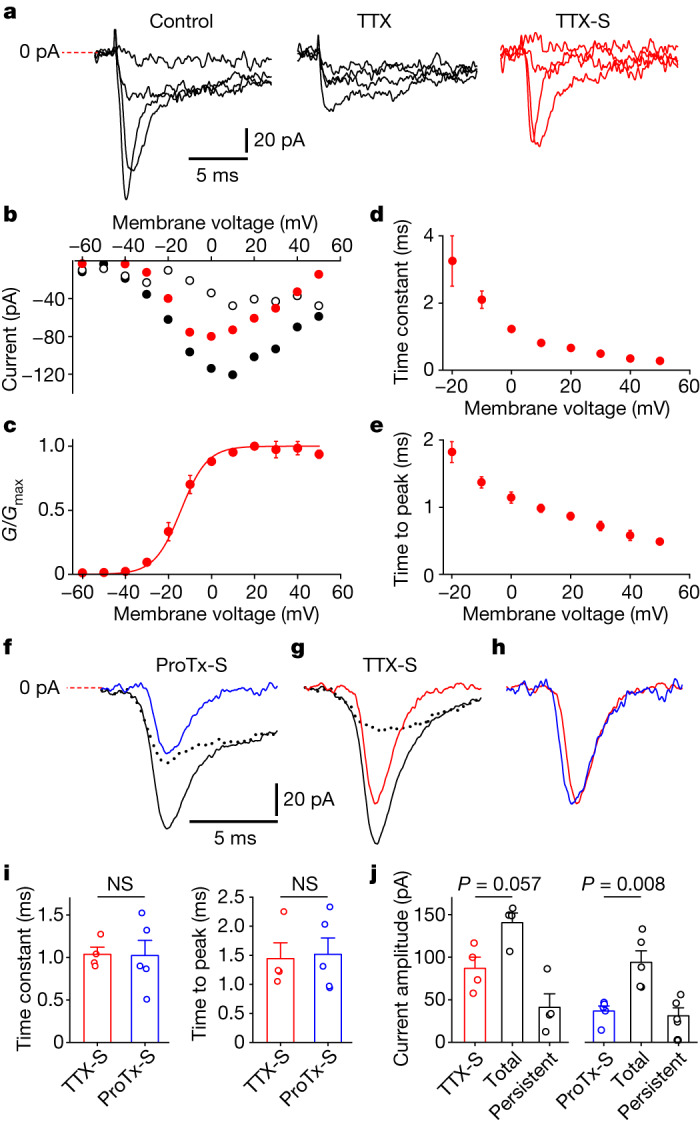
TTX-S currents are present in OA chondrocytes and are produced largely by Na_v_1.7 ProTx II-S channels. **a**, Representative current traces in control buffer (left), in the presence of 1 µM TTX (middle), and resulting traces of the TTX-S current (right). **b**, Current–voltage curves of peak current amplitudes in control solution () and in the presence of 1 µM TTX (), and the I-V curve for the TTX-S current (). **c**, *G*/*G*_max_ of the TTX-S current (mean ± s.e.m., *n* = 3) fitted with the Boltzmann equation. **d**, Single-exponential time constants of TTX-S current inactivation (mean ± s.e.m., *n* = 3). **e**, Time to peak of the TTX-S current (mean ± s.e.m., *n* = 3). **f**, Inhibition of fast-inactivating sodium current by 20 nM ProTx II. Averaged sodium current traces at 0 mV in control (black solid line) and in 20 nM ProTx II (black dotted line), and the resulting trace of their difference (ProTx II-S current, blue line). **g**, Inhibition of fast-inactivating sodium current by 1 µM TTX. Averaged traces of sodium currents evoked by 0 mV test voltage from −90 mV holding voltage in control solution (black solid line) and in the presence of 1 µM TTX (black dotted line), and the trace of their difference (TTX-S current, red trace). **h**, Overlays of TTX-S (red) and ProTx II-S (blue) current traces from **f**,**g**, normalized by peak current amplitudes. **i**, Left, inactivation time constants (mean ± s.e.m.) of TTX-S (*n* = 4) versus ProTx II-S (*n* = 5) currents. Right, time to peak of TTX-S (mean ± s.e.m., *n* = 4) versus ProTx II-S (mean ± s.e.m., *n* = 5) currents at 0 mV test voltage. **j**, Left, effect of 1 µM TTX on peak current amplitudes (mean ± s.e.m., *n* = 4) measured at 0 mV test voltage. Right, effect of 20 nM ProTx II on peak current amplitudes (mean ± s.e.m., *n* = 5) measured at 0 mV test voltage; averages of current amplitudes are shown for total, ProTx II-S and persistent sodium currents. *n* indicates cell number; *P* values by two-tailed Mann–Whitney test. NS, not significant.

We assessed the presence of Na_v_1.7 currents in 77 human chondrocytes isolated from three patients with OA (Supplementary Table 1). We first used 1 µM TTX, which blocks all Na_v_ channels except Na_v_1.5, Na_v_1.8 and Na_v_1.9, to isolate TTX-sensitive (TTX-S) currents which were obtained by subtraction of TTX-resistant (TTX-R) currents from currents in control solution at the respective voltages. Figure 1a shows representative current traces in response to −50 mV, −30 mV, −10 mV and 10 mV test pulses in control solution (Fig. 1a, left), in the presence of 1 µM TTX (Fig. 1a, middle), and the resulting traces of TTX-S current (Fig. 1a, right). Sodium currents were elicited by test voltages (−60 mV to 50 mV in 10 mV increments) applied from −90 mV holding potential. Because the current amplitude was relatively low, we enhanced the signal-to-noise ratio by averaging eight runs for each current–voltage trial. Consistent with a substantial contribution of Na_v_1.7, TTX-S current began to activate at −40 mV threshold, and exhibited maximal peak amplitude at 0 mV and sodium current reversal potential (*E*_r_) = −60.6 ± 2.3 mV (*n* = 3) (Fig. 1b). *G*/*G*_max_ of TTX-S current was calculated from current–voltage data, averaged at the respective membrane voltages (*n* = 3), and fitted by Boltzmann equation with voltage for half-maximal activation (*V*_1/2_) = −14.2 ± 0.8 mV, slope coefficient (*k*) = 6.1 ± 0.2 mV (*n* = 3) (Fig. 1c). On kinetic analysis, the falling phases of TTX-S current traces were best fitted with a single exponential. Inactivation time constants (single-exponential fits of the falling phase) were voltage-dependent, gradually decreasing with depolarizing membrane voltage from 3.3 ± 0.8 ms at −20 mV to 0.3 ± 0.1 ms (*n* = 3) at 50 mV membrane voltage (Fig. 1d). Time to peak of TTX-S current was measured from the voltage step onset and gradually decreased from 1.8 ± 0.2 ms at −20 mV to 0.5 ± 0.1 ms (*n* = 3) at 50 mV membrane voltage (Fig. 1e).

To further establish that the TTX-S currents included a Na_v_1.7 component, we compared the time course of the TTX-S and ProTx II-sensitive (ProTx II-S) currents. Inhibition by 1 µM TTX was observed in 4 out of 4 cells studied. Inhibition by 20 nM ProTx II, which selectively blocks Na_v_1.7, was seen in 5 out of 5 cells studied, confirming the presence of Na_v_1.7.

To assess the effect of ProTx II, we recorded sodium currents before (Fig. 1f, black solid trace), and after (Fig. 1f, black dotted trace) application of 20 nM ProTx II; each trace is an average of 15–30 consecutive sweeps with 2–3 s inter-sweep interval, and ProTx II-S current (Fig. 1f, blue) was obtained by point-by-point subtraction of current in the presence of ProTx II from the current recorded in control. Similarly, we obtained current sensitive to 1 µM TTX (Fig. 1g, red trace). Supporting the notion that the TTX-S currents were largely produced by Na_v_1.7, overlays of TTX-S (Fig. 1h, red trace) and ProTx II-S (Fig. 1h, blue trace) current traces normalized by peak current amplitudes showed similar time courses. Consistent with our observation of the similarities of TTX-S and ProTx II-S current time courses, their kinetic parameters were not significantly different. Inactivation time constants, obtained from a single-exponential fit of TTX-S and ProTx II-S currents elicited by 0 mV test voltage from −90 mV holding potential, were 1.0 ± 0.1 ms (*n* = 4) and 1.0 ± 0.2 ms (*n* = 5) (Fig. 1i), respectively, and their difference was not statistically significant (*P* > 0.05). Time-to-peak values of TTX-S and ProTx II-S currents at 0 mV membrane voltage were 1.4 ± 0.3 ms (*n* = 4) and 1.5 ± 0.3 ms (*n* = 5) (Fig. 1i), respectively, and were not statistically different (*P* > 0.05). These results add to the evidence that Na_v_1.7 contributes a substantial proportion of the Na^+^ current in OA chondrocytes. Total sodium current (average amplitude 141 ± 11 pA; *n* = 4) was inhibited by 1 µM TTX by 61.8 ± 16.4% (*n* = 4); TTX-S current amplitude at 0 mV was 87 ± 13 pA (*n* = 4) (Fig. 1j); and persistent (slow-inactivating, measured from control traces at 10 ms of test pulse onset) current amplitude was 41 ± 16 pA (*n* = 4). Total sodium current (93 ± 14 pA) was reduced by 20 nM ProTx II by 39.5 ± 13.5% (*n* = 5); ProTx II-S current amplitude at 0 mV was 37 ± 6 pA (*n* = 5); and persistent (slow-inactivating, measured from traces in control at 10 ms of test pulse onset) current amplitude was 31 ± 9 pA (*n* = 5) (Fig. 1j).

Together, these results establish the presence of Na_v_1.7 in OA chondrocytes by demonstrating that Na^+^ currents in these cells were sensitive to TTX, gating and kinetic properties similar to those known for Na_v_1.7, and inhibition by 20 nM ProTx II, a selective Na_v_1.7 blocker. The percentage of cells expressing Na_v_1.7 can be seen from our patch clamp recordings, which were obtained from 77 human OA chondrocytes. Inward sodium currents with a fast-inactivating (millisecond time constant at 0 mV test voltage; Fig. 1d) component and peak amplitude above 30 pA were evoked in 17% of cells. Average inward current amplitude at 0 mV test potential in these cells was 82 ± 19 pA (*n* = 13), and average current density was 2.4 ± 0.4 pA pF^−1^ (*n* = 13). A summary of the sodium current in human chondrocytes from three patients with OA is presented in Supplementary Table 1.

## Na_v_1.7 deletion in chondrocytes protects from OA

**Extended Data Fig. 2:**
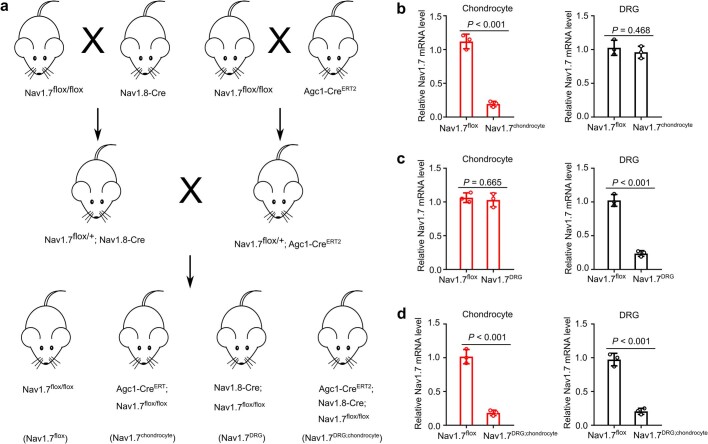
Generation of conditional Nav1.7 knockout mouse. **a**, Schematic of mouse breeding strategy to generate conditional knockout mice lacking Nav1.7 in chondrocyte (Nav1.7^chondrocyte^), DRG neurons (Nav1.7^DRG^) or both chondrocyte and DRG neurons (Nav1.7^DRG;chondrocyte^). **b**-**d**, Relative *Nav1.7* mRNA level in chondrocyte and DRG neurons from Nav1.7^chondrocyte^ (**b**), Nav1.7^DRG^ (**c**), and Nav1.7^DRG;chondrocyte^ (**d**) mice (n = 3 biological replicates). 10-week-old mice Agc1-Cre^ERT2^;Nav1.7^flox/flox^ and Agc1-Cre^ERT2^;Nav1.8-Cre;Nav1.7^flox/flox^ were intraperitoneally injected with tamoxifen at a dose of 150 µg per gram of body weight, administered daily for 5 consecutive days. Basal levels of Nav1.7 in DRG neurons and chondrocytes from Nav1.7^flox^ mice were set as 1, respectively. Data are means ± SD, *P* values are calculated by two-tailed unpaired Student’s t-test. Source Data

To examine the role of Na_v_1.7 in chondrocytes in OA and determine the relative contribution of DRG- and chondrocyte-expressed Na_v_1.7 to OA progression and pain, we generated mice with Na_v_1.7 knockout in DRG neurons (hereafter referred to as *Nav1.7*^*DRG*^; Na_v_1.7 is encoded by *Scn9a*), chondrocytes (hereafter referred to as *Nav1.7*^*chondrocyte*^), and both DRG neurons and chondrocytes (hereafter referred to as *Nav1.7*^*DRG;chondrocyte*^) by crossing Na_v_1.7-floxed (*Nav1.7*^*flox*^) mice with *Nav1.8*-*cre* (*Nav1.8* is encoded by *Scn10a*) mice and/or *Agc1*-*cre*^*ERT2*^ (*Agc1* is also known as *Acan*) mice, in which Cre-mediated recombination is induced by tamoxifen (Extended Data Fig. 2a). We confirmed Na_v_1.7 deletions in DRG and chondrocyte following tamoxifen administration in adult mice (Extended Data Fig. 2b–d).

**Fig. 2:**
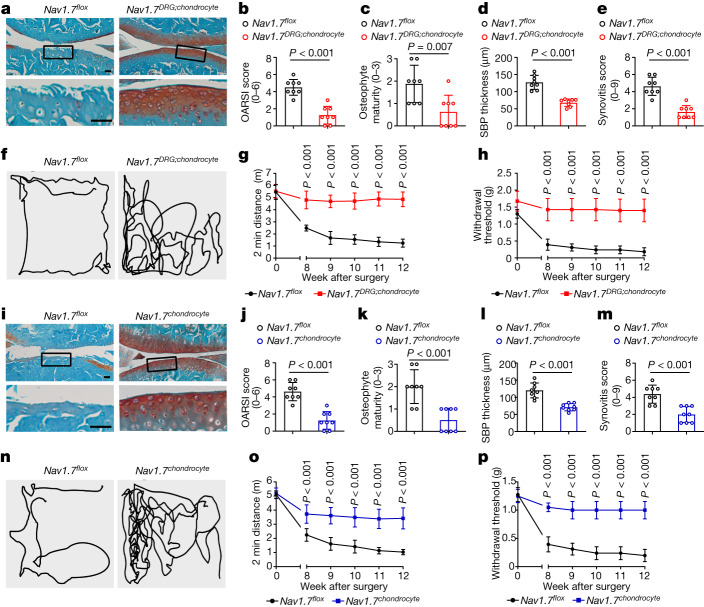
Ablation of chondrocyte Na_v_1.7 protects against OA and reduces pain. **a**, Safranin O and Fast Green-stained sections of knee joints of mice with the indicated genotype (*n* = 8). Scale bar, 50 µm. **b**–**e**, OARSI score (**b**), osteophyte development (**c**), subchondral bone plate (SBP) thickness (**d**) and synovitis score (**e**) in indicated mice 12 weeks after DMM (*n* = 8). **f**, Traces of open field testing at 12 weeks after DMM surgery. **g**,**h**, Two-minute travel distance (**g**) and von Frey testing (**h**) in DMM-operated mice at the indicated time points after surgery (*n* = 8). **i**, Safranin O and Fast Green-stained sections of knee joints (*n* = 8). Scale bars, 50 µm. **j**–**m**, OARSI score (**j**), osteophyte development (**k**), SBP thickness (**l**), and synovitis score (**m**) in indicated mice 12 weeks after DMM surgery (*n* = 8). **n**, Traces of open field testing at 12 weeks after DMM surgery. **o**,**p**, Two-minute travel distance (**o**) and von Frey testing (**p**) in DMM-operated mice at the indicated time points after surgery (*n* = 8). **b**–**e**,**j–m**, Data are mean ± s.d., *P* values by two-tailed unpaired Student’s *t*-test. **g**,**h**,**o**,**p**, Data are mean ± 95% confidence interval (CI), *P* values by two-tailed multiple unpaired Student’s *t*-test with Welch’s correction.

**Extended Data Fig. 3:**
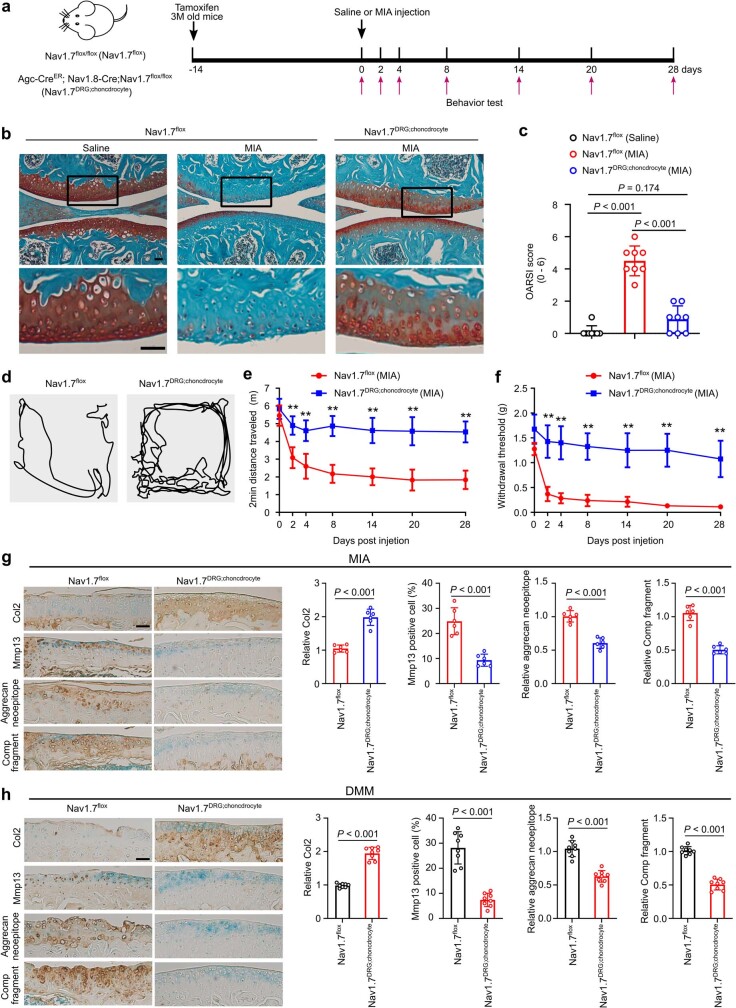
Genetic ablation of Nav1.7 in both chondrocyte and DRG neuron ameliorates OA progression and alleviates pain in chemically induced OA model. **a**, Experimental scheme showing establishment of MIA model in mice harboring tamoxifen inducible Nav1.7 deletion in chondrocyte and DRG neuron. **b**, Representative Safranin O/fast green stained knee joint sections of Nav1.7^flox^ and Nav1.7^DRG;chondrocyte^ male after saline or MIA injection at day 28 (n = 8 mice for each group). Scale bar = 50 µm. **c**, Quantification of OARSI score shown in **b**. **d**, Representative traces of open field testing at day 28 post MIA injection in Nav1.7^flox^ and Nav1.7^DRG;chondrocyte^ mice. **e**, **f**, Quantitation of 2 min travel distance (**e**) and von Frey testing (**f**) in Nav1.7^flox^ and Nav1.7^DRG^ mice at the indicated time-points post MIA injection (n = 8 mice per group). **g**, Immunohistochemical staining and corresponding quantification for COL2, Mmp13, Aggrecan neoepitope, and Comp fragment in knee joint sections of Nav1.7^flox^ and Nav1.7^DRG;chondrocyte^ mice at day 28 post MIA injection (n = 6). Scale bar = 50 µm**. h**, (**related to** Fig. 2a–h) Immunohistochemical staining and corresponding quantification of COL2, Mmp13, aggrecan neoepitope, and Comp fragment in knee joint sections of Nav1.7^flox^ and Nav1.7^DRG; chondrocyte^ mice at 12 weeks post DMM surgery (n = 8). Scale bar = 50 µm. Data are means ± SD, *P* values are calculated by one way ANOVA with Bonferroni post-hoc test (**c**) and two-tailed unpaired Student’s t-test (**g**, **h**). Data are means ± 95% confidence interval (CI), *P* values are calculated by two-tailed multiple unpaired Student’s t-test with Welch correction (**e, f**). * *P* < 0.05; ** *P* < 0.01. Source Data

The two most common methods to experimentally model OA in mice include surgical and chemical induction. As in humans, both surgically and chemically induced mouse OA models exhibit articular cartilage erosion or loss and OA-related pain^23,24^. We established both surgically induced destabilization of the medial meniscus (DMM) (Fig. 2) and chemically induced MIA models (Extended Data Fig. 3a) in *Nav1.7*^*flox*^ and *Nav1.7*^*DRG;chondrocyte*^ mice. Histological analysis revealed that Na_v_1.7 deletion in both DRG neurons and chondrocytes substantially attenuated cartilage loss, and reduced the Osteoarthritis Research Society International (OARSI) score (Fig. 2a,b and Extended Data Fig. 3b,c) in both DMM and MIA OA models. Notably, *Nav1.7*^*DRG;chondrocyte*^ mice exhibited markedly reduced osteophyte formation, thickening of subchondral bone plate, suggestive of sclerosis, and decreased synovitis score in the DMM model (Fig. 2c–e). We evaluated the association of Na_v_1.7 with OA pain by measuring open field movement activity and mechanical allodynia with von Frey testing. *Nav1.7*^*DRG;chondrocyte*^ mice exhibited greater overall distance of movement and significantly reduced mechanical allodynia throughout the three-month period after DMM surgery and the four-week period after MIA injection relative to *Nav1.7*^*flox*^ mice (Fig. 2f–h and Extended Data Fig. 3d–f). Immunohistochemical staining of knee joints indicated that OA-associated loss of the anabolic marker type II collagen (COL2), and increase of MMP13, aggrecan neoepitope generated via cleavage by ADAMTS5, and COMP fragment observed in *Nav1.7*^*flox*^ mice, were inhibited in *Nav1.7*^*DRG;chondrocyte*^ mice in both surgically and chemically induced OA (Extended Data Fig. 3g,h). These findings indicate that chondrocyte- and DRG neuron- expressed Na_v_1.7 concurrently contribute to modulating OA progression and OA-associated pain.

**Extended Data Fig. 4:**
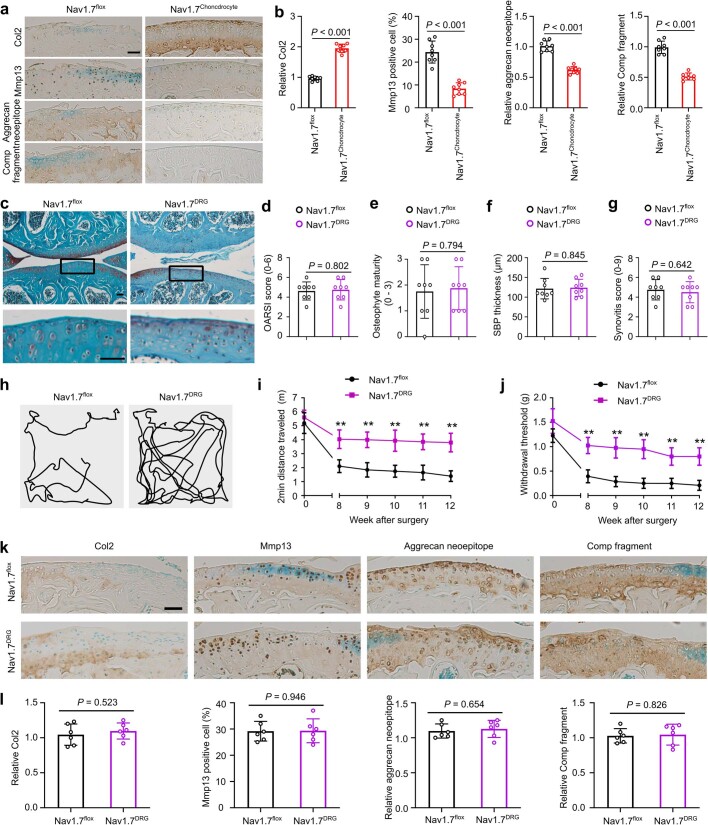
Genetic ablation of Nav1.7 in DRG neuron contributes to reduce OA associated pain without affecting OA pathological progression in surgically induced OA model. **a**, **b**, (**related to** Fig. 2i–p) Immunohistochemical staining (**a**) and corresponding quantification (**b**) of Col2, Mmp13, Aggrecan neoepitope, and Comp fragment in knee joint sections of Nav1.7^flox^ and Nav1.7^Chondrocyte^ mice at 12 weeks post DMM surgery (n = 8). Scale bar = 50 µm in **a**. **c**, Representative Safranin O/fast green stained sections of knee joints from DMM operated Nav1.7^flox^ and Nav1.7^DRG^ mice (n = 8 mice per group). Scale bar = 50 µm. **d**-**g**, Quantitation of OARSI score (**d**), osteophyte development (**e**), SBP thickness (**f**), and synovitis score (**g**) in Nav1.7^flox^ and Nav1.7^DRG^ mice subjected to DMM surgery at week 12 (n = 8 mice per group). **h**, Representative traces of open field testing at 12 weeks post DMM surgery in Nav1.7^flox^ and Nav1.7^DRG^ mice. **i**, **j**, Quantitation of 2 min travel distance (**i**) and von Frey testing (**j**) in DMM operated Nav1.7^flox^ and Nav1.7^DRG^ mice at the indicated time-points after surgery (n = 8 mice per group). **k**, **l**, Immunohistochemical staining (**k**) and corresponding quantification (**l**) for Col2, Mmp13, Aggrecan neoepitope, and Comp fragment in knee joint sections of Nav1.7^flox^ and Nav1.7^DRG^ mice at 12 weeks post DMM surgery (n = 6). Scale bar = 50 µm in **k**. Data are means ± SD, *P* values are calculated by two-tailed unpaired Student’s t-test in b, d-g and l; data are means ± 95% confidence interval (CI), *P* values are calculated by two-tailed multiple unpaired Student’s t-test with Welch correction in i and j (** *P* < 0.01). Source Data

To distinguish the contributions of Na_v_1.7 expressed by chondrocytes and DRG neurons towards progression of OA pathology and OA-associated pain, we established the DMM OA model in *Nav1.7*^*flox*^ and *Nav1.7*^*chondrocyte*^ or *Nav1.7*^*DRG*^ mice. Similar to our observations in *Nav1.7*^*DRG;chondrocyte*^ DMM mice, *Nav1.7*^*chondrocyte*^ mice also displayed substantial reductions in DMM-induced cartilage loss with reduced osteophyte formation, subchondral bone plate thickening and synovitis score compared with *Nav1.7*^*flox*^ mice (Fig. 2i–m). Deletion of Na_v_1.7 in chondrocytes reduced the loss of open field movement activity and mechanical allodynia (von Frey test) in the DMM model (Fig. 2n–p). In addition, deletion of Na_v_1.7 in chondrocytes increased the amount of the anabolic effector COL2 and simultaneously decreased the amount of DMM-induced catabolic effectors, including MMP13, aggrecan neoepitope and COMP fragment (Extended Data Fig. 4a,b). By contrast, deletion of Na_v_1.7 in DRG neurons reduced DMM-induced pain without affecting structural abnormalities such as cartilage destruction, osteophyte formation, subchondral bone plate thickening and synovitis score (Extended Data Fig. 4c–j). Immunohistochemical staining revealed that there was no difference between *Nav1.7*^*flox*^ and *Nav1.7*^*DRG*^ DMM mice in terms of the amounts of COL2, MMP13, aggrecan neoepitope and COMP fragment in cartilage (Extended Data Fig. 4k,l), supporting the notion that Na_v_1.7 deletion in DRG neurons is not involved in the regulation of cartilage loss. In sum, Na_v_1.7 expressed in chondrocytes modulates OA progression and resultant OA-associated pain behaviour, whereas Na_v_1.7 expressed by DRG neurons contributes to OA-associated pain without affecting OA progression.

## Na_v_1.7 inhibition protects against OA

**Extended Data Fig. 5:**
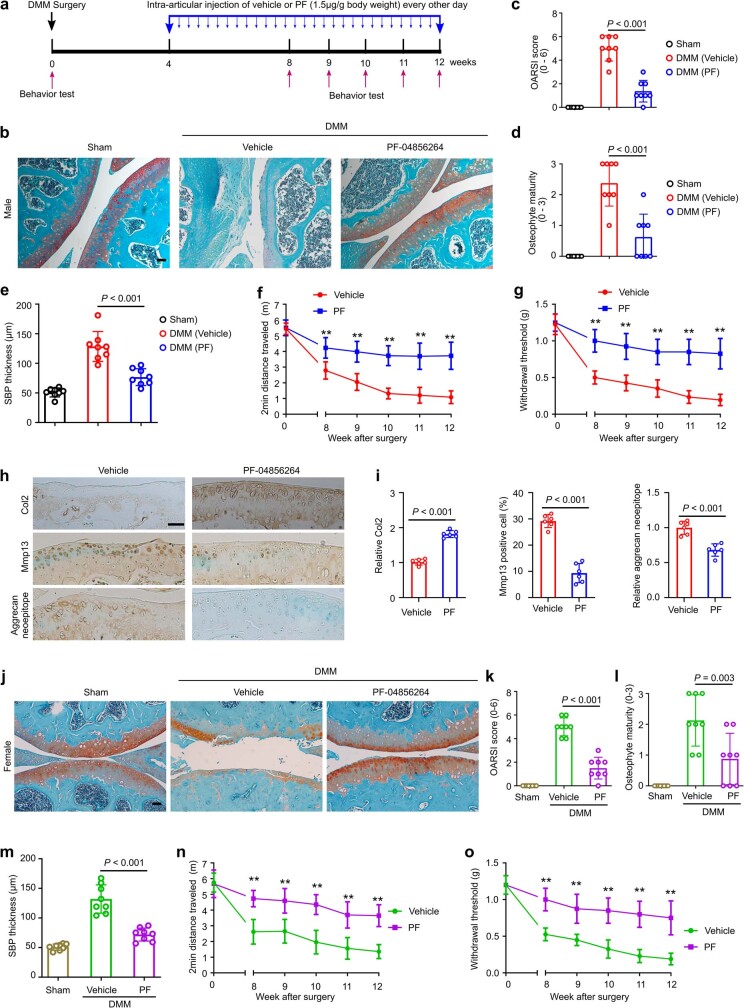
Pharmacological blockade of Nav1.7 through locally is therapeutic against OA and OA-related pain in surgically induced mouse models. **a**, Schematic of the experimental design to determine the effects of intra-articular administrated PF-04856264 (the selective Nav1.7 inhibitor) on OA progression and pain-related behaviors in the surgically induced DMM mouse model. **b**, Representative Safranin O/fast green stained knee joint sections of DMM operated WT male mice treated with or without PF-04856264 for 8 weeks (n = 8 mice for each group). Scale bar = 50 µm. **c**-**e**, Quantitation of OARSI score (**c**), osteophyte development (**d**) and SBP thickness (**e**) in DMM operated WT male mice treated with or without PF-04856264 for 8 weeks (n = 8 mice per group). **f**, **g**, Quantitation of 2 min travel distance (**f**) and von Frey testing (**g**) in DMM-operated WT male mice treated with or without PF-04856264 at the indicated time-points after surgery (n = 8 mice per group). **h**, **i**, Immunohistochemical staining (**h**) and corresponding quantification (**i**) of Col2, Mmp13, and Aggrecan neoepitope in knee joint sections of DMM operated WT male mice treated with or without PF-04856264 for 8 weeks (n = 6). Scale bar = 50 µm in **h**. **j**, Representative Safranin O/fast green stained knee joint sections of DMM operated WT female mice treated with or without PF-04856264 for 8 weeks (n = 8 mice for each group). Scale bar = 50 µm. **k**-**m**, Quantitation of OARSI score (**k**), osteophyte development (**l**) and SBP thickness (**m**) in DMM operated WT female mice treated with or without PF-04856264 for 8 weeks (n = 8 mice per group). **n**, **o**, Quantitation of 2 min travel distance (**n**) and von Frey testing (**o**) in DMM-operated WT female mice treated with or without PF-04856264 at the indicated time-points after surgery (n = 8 mice per group). Data are means ± SD, *P* values are calculated by one way ANOVA with Bonferroni post-hoc test (**c**, **d**, **e**, **k**, **l** and **m**) and two-tailed unpaired Student’s t-test (**i**). Data are means ± 95% confidence interval (CI), *P* values are calculated by two-tailed multiple unpaired Student’s t-test with Welch correction in f, g, n and o (** *P* < 0.01). Source Data

We then sought to determine whether pharmacological Na_v_1.7 blockade with PF-04856264, a selective Na_v_1.7 blocker^25^, could inhibit cartilage destruction and alleviate pain. To this end, we first established the DMM OA model in 12-week-old C57BL/6 wild-type male mice, followed by intra-articular injection of vehicle or PF-04856264 at 1.5 µg per g body weight every other day beginning 4 weeks post-operatively for a total of 8 weeks (Extended Data Fig. 5a). Safranin O staining revealed that PF-04856264 treatment protected the structure of articular cartilage and maintained proteoglycan content in the cartilage compared with vehicle (Extended Data Fig. 5b). PF-04856264 treatment was associated with significantly lower OARSI scores, osteophyte development and subchondral bone plate thickness relative to vehicle treatment (Extended Data Fig. 5c–e). In addition, DMM-induced OA pain behaviour was reduced and COL2 level was increased in mice treated with PF-04856264 (Extended Data Fig. 5f–h). Accordingly, Col2 level was increased; by contrast, MMP13 and aggrecan neoepitope levels were significantly reduced in the cartilage of mice with DMM OA that received intra-articular injections of PF-04856264 compared with those treated with vehicle (Extended Data Fig. 5h,i). To explore potential sex differences, we replicated the DMM OA model in female wild-type mice using the same treatment approach. Consistent with the observations in male mice, female mice treated with PF-04856264 exhibited less articular cartilage loss, lower OARSI score and significant reductions in pain behaviour compared with untreated controls (Extended Data Fig. 5j–o).

**Extended Data Fig. 6:**
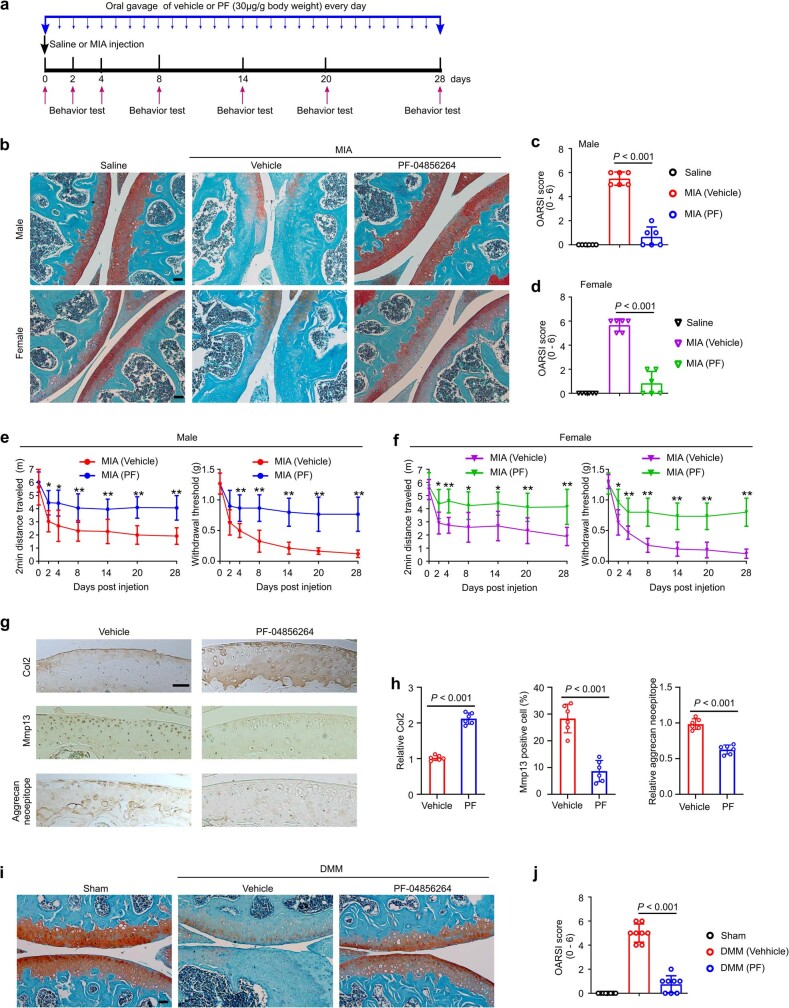
Oral delivery of PF-04756264 attenuates OA progression and alleviates pain in both chemically and surgically induced OA models. **a**, A schematic representation highlights the experimental outline to determine the effects of PF-04856264 through oral delivery on OA progression and behavior changes in WT male and female mice with MIA model. **b**, Representative Safranin O/fast green stained knee joint sections of WT male and female mice treated with or without PF-04856264 after saline or MIA injection at day 28 (n = 6 mice for each group). Scale bar = 50 µm. **c**, **d**, Quantification of OARSI score in male (**c**) and female (**d**) mice as shown in **b**. **e**, **f**, Quantitation of 2 min travel distance and von Frey testing in WT male (**e**) and female (**f**) mice treated with or without PF-04856264 at the indicated time-points post MIA injection (n = 6 mice per group). **g**, **h**, Immunohistochemical staining (**g**) and corresponding quantification (**h**) for Col2, Mmp13, and Aggrecan neoepitope in knee joint sections of WT male mice treated with or without PF-04856264 at day 28 post MIA injection (n = 6). Scale bar = 50 µm in **g**. **i**, Representative Safranin O/fast green stained knee joint sections of DMM operated WT male mice orally treated without or with PF-04856264 for 8 weeks (n = 8 mice for each group). Scale bar = 50 µm. **j**, Quantitation of OARSI score for **i**. Data are means ± SD, *P* values are calculated by one way ANOVA with Bonferroni post-hoc test (**c**, **d** and **j**) and two-tailed unpaired Student’s t-test (**h**). Data are means ± 95% confidence interval (CI), *P* values are calculated by two-tailed multiple unpaired Student’s t-test with Welch correction in e and f (* *P* < 0.05; ** *P* < 0.01). Source Data

To further demonstrate the effectiveness of Na_v_1.7 blockade in preventing OA, we established the MIA model in wild-type male and female mice (Extended Data Fig. 6a). Since the route of drug delivery largely determines the drug concentrations achieved at the target site and thus therapeutic efficacy, and because systemic delivery is well accepted by patients, we determined the therapeutic effects of systemic administration of PF-04856264 through oral gavage at 30 µg per g body weight in the MIA model (Extended Data Fig. 6a). Similar to the results from local delivery, systemic delivery of PF-04856264 also markedly slowed OA progression as demonstrated by less cartilage loss, and improved OARSI score compared to vehicle treatment (Extended Data Fig. 6b–d). Furthermore, systemic PF-04856264 substantially increased the distance of movement in the open field test and von Trey hindpaw withdrawal threshold (Extended Data Fig. 6e,f). No apparent sex differences were observed, as PF-04856264 elicited cartilage loss protection and pain relief in both male and female mice (Extended Data Fig. 6b–f). Immunohistochemical staining revealed that oral PF-04856264 delivery increased COL2 expression and decreased MMP13 and aggrecan neoepitope levels in cartilage compared with vehicle (Extended Data Fig. 6g,h). Oral delivery of PF-04856264 also reduced cartilage loss in mice with DMM-induced OA (Extended Data Fig. 6i,j).

## CBZ reduces cartilage loss and pain in OA

**Extended Data Fig. 7:**
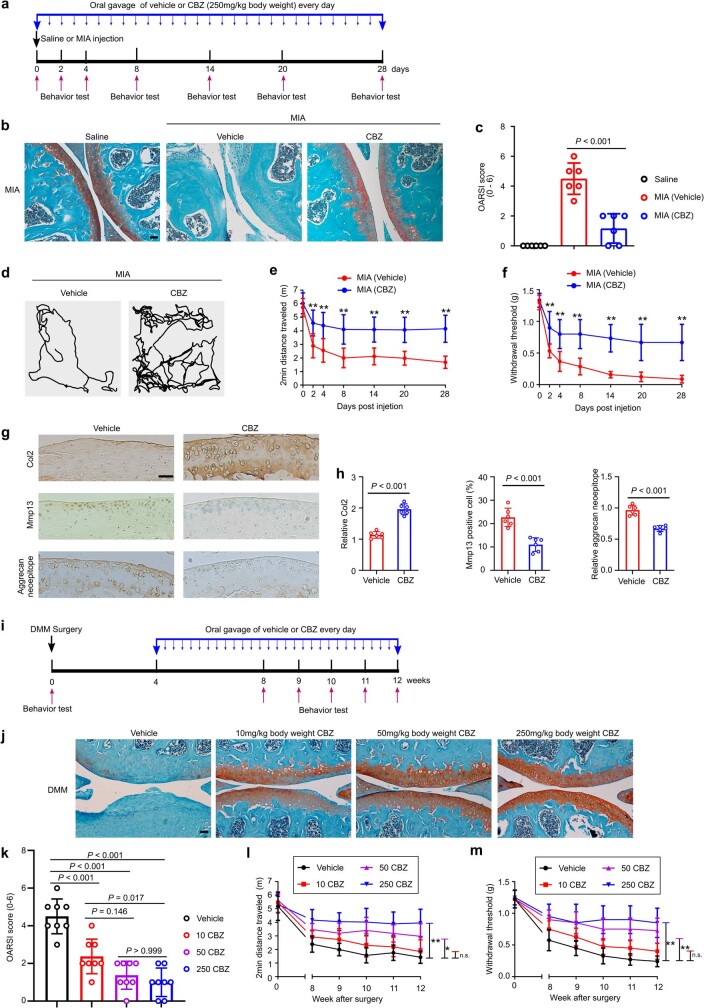
Carbamazepine (CBZ), a clinically used sodium chancel blocker, attenuates OA progression and pain in chemically and surgically induced OA models. **a**, Schematic of timeline to analyze the effects of systemic oral delivery carbamazepine (CBZ) on OA progression and pain in MIA model. **b**, Representative Safranin O/fast green stained knee joint sections of WT male mice treated with or without CBZ after saline or MIA injection at day 28 (n = 6 mice for each group). Scale bar = 50 µm. **c**, Quantification of OARSI score as shown in **b**. **d**, Representative traces of open field testing at day 28 post MIA injection in WT male mice with indicated treatment at day 28 post MIA injection. **e**, **f**, Quantitation of 2 min travel distance (**e**) and von Frey testing (**f**) in WT male mice at the indicated time-points post MIA injection (n = 6 mice per group). **g**, **h**, Immunohistochemical staining (**g**) and corresponding quantification (**h**) for Col2, Mmp13, and Aggrecan neoepitope in knee joint sections of WT mice with indicated treatment at day 28 post MIA injection (n = 6). Scale bar = 50 µm in **g**. **i**, Schematic of timeline to analyze the effects of systemic oral delivery of various dosages of CBZ on OA progression and pain in surgically induced DMM model. **j**, Representative Safranin O/fast green stained knee joint sections of DMM operated WT male mice treated without or with 10 mg/kg body weight, 50 mg/kg body weight or 250 mg/kg body weight CBZ for 8 weeks (n = 8 mice for each group). Scale bar = 50 µm. **k**, Quantitation of OARSI score for **j**. **l**, **m**, Quantitation of 2 min travel distance (**l**) and von Frey testing (**m**) in DMM-operated WT male mice treated without or with various dosage of CBZ, as indicated, at specified time-points after surgery (n = 8 mice per group). Data are means ± SD, *P* values are calculated by one way ANOVA with Bonferroni post-hoc test (**c**, **k**) and two-tailed unpaired Student’s t-test (**h**). Data are means ± 95% confidence interval (CI), *P* values are calculated by two-tailed multiple unpaired Student’s t-test with Welch correction in (**e** and **f**) and two way ANOVA with Bonferroni post-hoc test (**l, m**). * *P* < 0.05; ** *P* < 0.01.

Carbamazepine (CBZ) is a clinically used Na channel inhibitor that is known to act on Na_v_1.7 (ref. ^26^) and has been approved by the US Food and Drug Administration (FDA) for indications in epilepsy, bipolar disorder and neuropathic pain. To determine whether this sodium channel inhibitor has therapeutic effects on OA, we systemically delivered CBZ into MIA mice by oral gavage (250 mg per kg body weight, daily)—equivalent to the dosage used to treat epilepsy in humans, taking species differences into account^27^, and suggested to alleviate disease severity in a metaphyseal chondrodysplasia Schmid type mouse model^28^ (Extended Data Fig. 7a). Systemic CBZ treatment closely recapitulated the therapeutic effects of systemic PF-04856264 treatment on OA in terms of attenuation of cartilage loss, alleviation of OA pain and protection against changes in pro-anabolic and anti-catabolic effects in chondrocytes by upregulating COL2 expression and downregulating the expression of matrix-degrading enzymes (Extended Data Fig. 7b–h). We also evaluated the therapeutic effects of varying doses of CBZ administered orally in the DMM model (Extended Data Fig. 7i). In mice that underwent DMM surgery, a low dose of CBZ (10 mg per kg body weight) led to a significant reduction in cartilage loss 12 weeks after surgery. However, there was no significant difference in pain behaviour compared with untreated DMM mice (Extended Data Fig. 7j–m). Medium (50 mg per kg body weight) and high (250 mg per kg body weight) doses of CBZ provided stronger protection against cartilage loss and significantly reduced OA-associated pain behaviour compared with the low dose (Extended Data Fig. 7j–m). These findings suggest that non-specific VGSC blockers such as CBZ may hold promise as novel disease-modifying treatments for OA, and do not simply act in an analgesic manner.

## Na_v_1.7 blockade regulates chondrocyte biology

**Extended Data Fig. 8:**
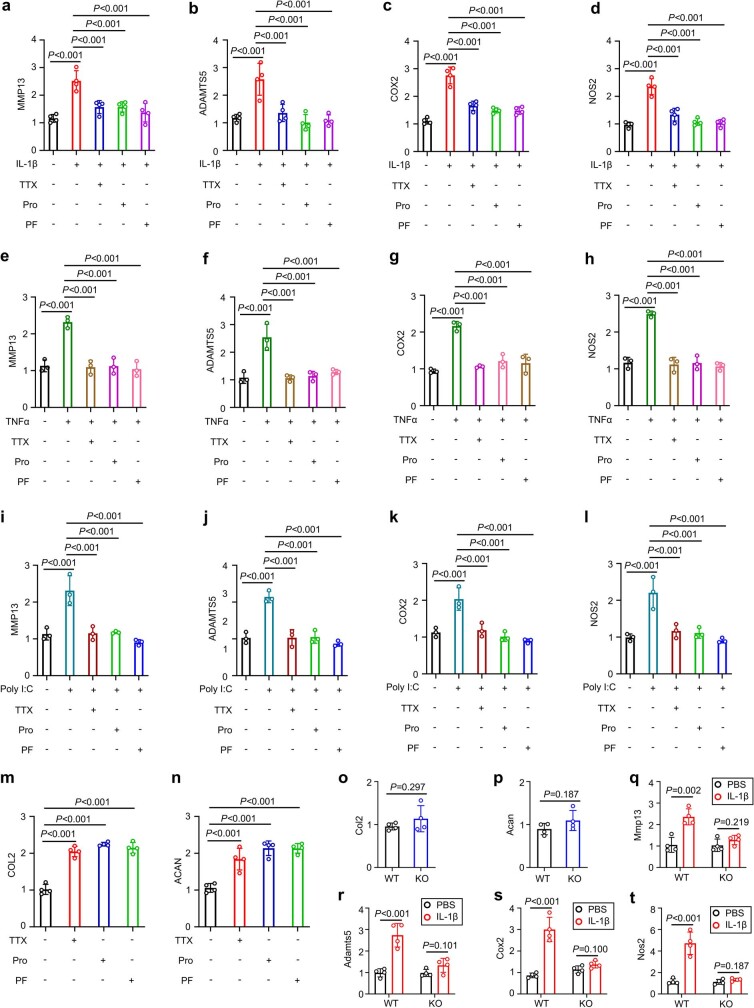
Pharmacological blockade or deletion of Nav1.7 enhances anabolic and inhibits catabolic metabolism in chondrocytes. **a**-**d**, mRNA levels of catabolism markers *MMP13* (**a**), *ADAMTS5* (**b**), *COX2* (**c**) and *NOS2* (**d**) in human C28I2 chondrocytes treated with or without 10 ng/ml IL-1β in the absence or presence of 1 µM TTX, 25 nM ProTx II (Pro) or 1 µM PF-04856264 (PF) for 24 h, assayed by qRT-PCR (n = 4 biological replicates). **e**-**h**, mRNA levels of catabolism markers *MMP13* (**e**), *ADAMTS5* (**f**), *COX2* (**g**) and *NOS2* (**h**) in human C28I2 chondrocytes treated with or without 10 ng/ml TNFα in the absence or presence of 1 µM TTX, 25 nM ProTx II (Pro) or 1 µM PF-04856264 (PF) for 24 h, assayed by qRT-PCR (n = 3 biological replicates). **i**-**l**, mRNA levels of catabolism markers *MMP13* (**i**), *ADAMTS5* (**j**), *COX2* (**k**) and *NOS2* (**l**) in human C28I2 chondrocytes treated with or without 500 ng/ml poly(I:C) in the absence or presence of 1 µM TTX, 25 nM ProTx II (Pro) or 1 µM PF-04856264 (PF) for 24 h, assayed by qRT-PCR (n = 3 biological replicates). **m**, **n**, mRNA levels of anabolic markers *COL2* (**m**) and *ACAN* (**n**) in human C28I2 chondrocytes treated with 1 µM TTX, 25 nM ProTx II (Pro) or 1 µM PF-04856264 (PF) for 24 h, assayed by qRT-PCR (n = 4 biological replicates). **o**, **p**, mRNA levels of *Col2* (**o**) and *Acan* (**p**) in chondrocytes isolated from Nav1.7^flox^ and Nav1.7^chondrocyte^ mice at P6 (n = 4 biological replicates). **q**-**t**, mRNA levels of *Mmp13* (**q**), *Adamts5* (**r**), *Cox2* (**s**) and *Nos2* (**t**) in chondrocytes isolated from Nav1.7^flox^ and Nav1.7^chondrocyte^ mice at P6 which are treated with or without IL-1β for 24 h (n = 4 biological replicates). Data are mean ± SD, *P* values are calculated by one way ANOVA with Bonferroni post-hoc test (**a**-**n**) and two-tailed unpaired Student’s t-test (**o**-**t**).

Given that Na_v_1.7 blockade led to pro-anabolic and anti-catabolic effects on chondrocytes in OA models, we next sought to determine whether Na_v_1.7 blockade would affect chondrocyte biology in vitro. Expression of genes encoding catabolic molecules induced by IL-1β, including *MMP13*, *ADAMTS5*, *COX2* (also known as *PTGS2*) and *NOS2*, were inhibited by 1 µM TTX (Extended Data Fig. 8a–d). To further demonstrate the importance of Na_v_1.7 in chondrocyte biology, we used Na_v_1.7-selective blockers and a loss-of-function Na_v_1.7 mutant approach. We used ProTx II^29^ (half-maximal inhibitory concentration (IC_50_) = 0.3 nM) and PF-04856264 (ref. ^25^) (IC_50_ = 28 nM) to inhibit Na_v_1.7 pharmacologically. Similar to TTX, blockade of Na_v_1.7 with 25 nM ProTx II or 1 µM PF-04856264 (Extended Data Fig. 8a–d) demonstrated the importance of Na_v_1.7 in inhibiting IL-1β-induced catabolism. To further assess whether Na_v_1.7 blockade effectively inhibits chondrocyte catabolism in an inflammatory OA environment in general, we evaluated the effects of these inhibitors on chondrocyte catabolism in the presence of the additional inflammatory stimuli TNF^30^ and polyinosinic–polycytidilic acid^31^ (poly(I:C)). Simultaneous treatment of Na_v_1.7 blockers significantly inhibited TNF- and poly(I:C)-induced chondrocyte catabolism (Extended Data Fig. 8e–l). VGSC blockade with TTX, ProTx II or PF-04856264 also significantly induced the expressions of genes encoding anabolic molecules such as *COL2* and *ACAN* (Extended Data Fig. 8m,n). We also isolated chondrocytes from *Nav1.7*^*flox*^ and *Nav1.7*^*chondrocyte*^ mice, and found that Na_v_1.7 deletion blocked IL-1β-induced catabolism. Notably, Na_v_1.7 deletion did not change anabolism under physiological conditions (Extended Data Fig. 8o–t).

**Extended Data Fig. 9:**
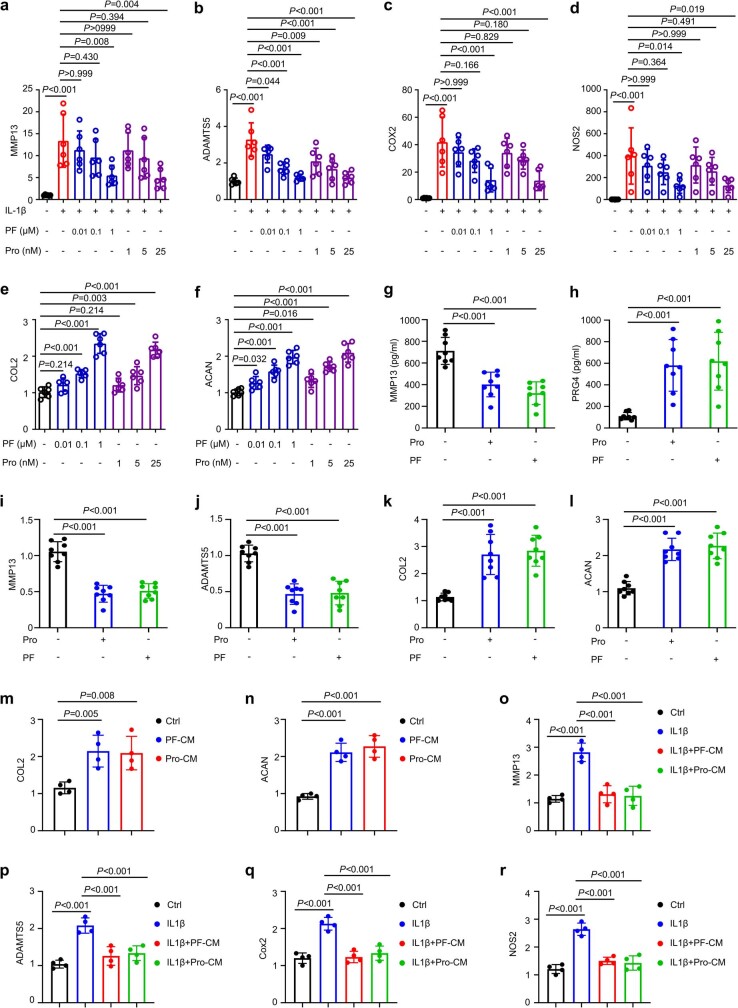
Blocking Nav1.7 pharmacologically inhibit catabolism and enhance anabolism through regulating the chondrocyte secretion. **a**-**d**, mRNA levels of *MMP13* (**a**), *ADAMTS5* (**b**), *COX2* (**c**) and *NOS2* (**d**) in primary human chondrocytes isolated from patients with last stage OA which are treated with or without 10 ng/ml IL-1β along with a serial doses of ProTx II (Pro) or PF-04856264 (PF) for 24 h, assayed by qRT-PCR (n = 6 donors). **e**, **f**, mRNA levels of *COL2* (**e**) and *ACAN* (**f**) in primary human chondrocytes isolated from patients with late stage OA which are treated with a serial doses of ProTX II (Pro) or PF-04856264 (PF) for 24 h, assayed by qRT-PCR (n = 6 donors). **g**, **h**, Proteins levels of MMP13 (**g**) and PRG4 (**h**) in the supernatants of full-thickness human OA cartilage explants, as determined by ELISA (n = 8 donors). Cartilage explant was cultured with 10 ng/ml IL-1β in the absence or presence of 25 nM ProTx II or 1 µM PF-04856264 for 5 days. **i**-**l**, mRNA levels of *MMP13* (**i**), *ADAMTS5* (**j**), *COL2* (**k**), and *ACAN* (**l**) in the human OA cartilage explants cultured with 10 ng/ml IL-1β in the absence or presence of 25 nM ProTx II or 1 µM PF-04856264 for 5 days, assayed by qRT-PCR (n = 8 donors). **m**, **n**, mRNA levels of *COL2* (**m**) and *ACAN* (**n**) in human C28I2 cells treated with conditioned medium collected from 25 nM Pro or 1 µM PF treated C28I2 cells (n = 4 biological replicates). **o**-**r**, mRNA levels of *MMP13* (**o**), *ADAMTS5* (**p**), *COX2* (**q**) and *NOS2* (**r**) in human C28I2 cells treated with conditioned medium collected from 25 nM Pro or 1 µM PF treated C28I2 cells as well as 10 ng/ml IL-1β for 24 h (n = 4 biological replicates). Data are mean ± SD, *P* values are calculated by one way ANOVA with Bonferroni post-hoc test.

To establish the relevance of our findings in a human context, we characterized the effects of Na_v_1.7 on primary human chondrocytes isolated from six patients with KL 3–4 knee OA. We confirmed that both ProTx II and PF-04856264 decreased IL-1β-induced expression of *MMP13*, *ADAMTS5*, *COX2* and *NOS2* (Extended Data Fig. 9a–d) and increased the expression of *COL2* and *ACAN* (Extended Data Fig. 9e,f). To further determine the effects of Na_v_1.7 blockers on human OA cartilage, we used an ex vivo cartilage explant assay using full-thickness OA cartilage from tibia plateaus of patients with OA who were undergoing total knee arthroplasty. In line with the results obtained from in vitro monolayer culture, supernatants of explants treated with Na_v_1.7 blockers contained significantly lower levels of the cartilage catabolic marker MMP13 and higher levels of cartilage matrix component lubricin^32^ (also known as proteoglycan 4 (PRG4)) compared with vehicle-treated controls (Extended Data Fig. 9g,h). In addition, Na_v_1.7 blockers decreased the expression of catabolic markers *MMP13* and *ADAMTS5*, and increased the expression of anabolic markers *COL2* and *ACAN* in human OA cartilage explants cultured under inflammatory conditions (Extended Data Fig. 9i–l).

VGSCs are transmembrane proteins that open when the membrane potential in their vicinity becomes depolarized. There is increasing evidence that dynamic membrane potential influences a wide range of biological functions in both excitable and non-excitable cells; regulated secretion is one such function that has been widely studied^33,34^. In light of these observations, we hypothesized that alterations in secretion or cross-membrane transport of proteins might contribute to the regulation of chondrocyte biology induced by Na_v_1.7 inhibition. We examined whether incubation with conditioned medium collected from chondrocytes treated with Na_v_1.7-selective inhibitors induced the same regulatory effects on chondrocyte biology observed with Na_v_1.7 inhibition. We found that conditioned medium collected from chondrocytes treated with PF-04856264 or ProTx II promoted the expression of anabolic markers and abolished IL-1β-induced expression of catabolic molecules, similar to direct treatment of chondrocytes with these Na_v_1.7 inhibitors (Extended Data Fig. 9m–r). These findings suggest that secreted molecules in conditioned medium are responsible for the effects of Na_v_1.7 blockade on chondrocyte biology.

**Extended Data Fig. 10:**
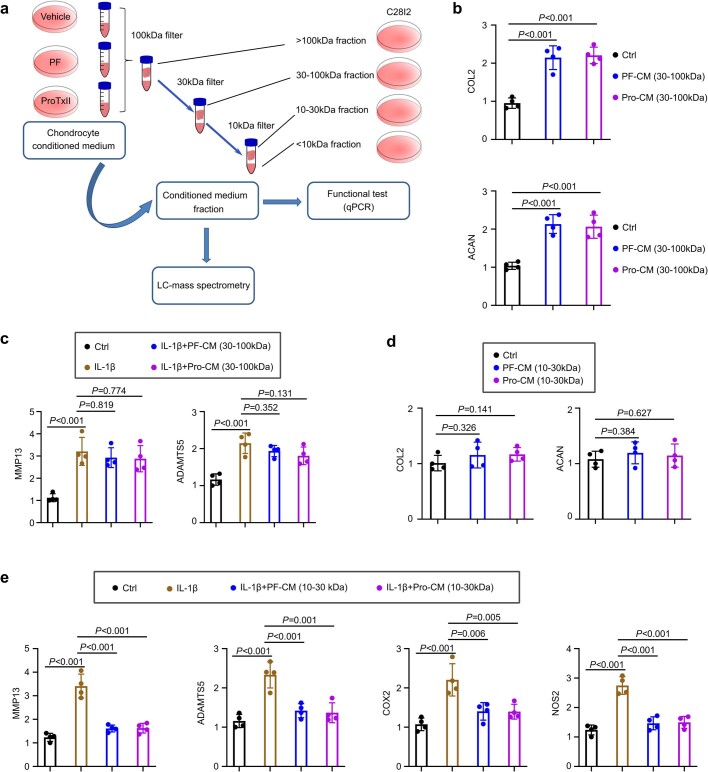
Effects of conditioned medium on human chondrocyte anabolism and catabolism. **a**, Schematic of the experimental strategy of conditioned medium production, fraction and characterization. Human C28I2 chondrocytes are expanded in DMEM supplemented with FBS until they are 90% confluent. The growth medium is them exchanged with medium supplemented with ITS and 25 nM ProTX II (Pro) or 1 µM PF-04856264 (PF) for 2 days. The medium is then collected and separated into 4 fractions based on sized exclusion: <10 kDa, 10−30 kDa, 30−100 kDa, and >100 kDa using the centrifugal filter device. **b**, mRNA levels of *COL2 and ACAN* in human C28I2 cells treated with 30–100 kDa fraction of the conditioned medium for 24hrs (n = 4 biological replicates). **c**, mRNA levels of *MMP13* and *ADAMTS5* in human C28I2 cells treated with 30–100 kDa fraction of the conditioned medium in the absence or presence of 10 ng/ml IL-1β for 24hrs (n = 4 biological replicates). **d**, mRNA levels of *COL2* and *ACAN* in human C28I2 cells treated with 10–30 kDa fraction of the conditioned medium for 24hrs (n = 4 biological replicates). **e**, mRNA levels of *MMP13, ADAMTS5, COX2* and *NOS2* in human C28I2 cells treated with 10–30 kDa fraction of the conditioned medium in the absence or presence of 10 ng/ml IL-1β for 24hrs (n = 4 biological replicates). Data are means ± SD, *P* values are calculated by one way ANOVA with Bonferroni post-hoc test (**b**-**e**). Source Data

We next attempted to isolate and characterize the molecules in conditioned medium that mediate Na_v_1.7 regulation of chondrocyte anabolism and catabolism. To this end, we separated the components of conditioned medium into 4 fractions based on sized exclusion: <10 kDa, 10–30 kDa, 30–100 kDa and over 100 kDa, and tested their effects on chondrocyte biology (Extended Data Fig. 10a). We found that the 30–100 kDa fraction was capable of promoting chondrocyte anabolism without affecting catabolism, whereas the 10–30 kDa fraction only inhibited cytokine-induced catabolism (Extended Data Fig. 10b–e). These findings suggest that one or more secreted proteins in the 30–100 kDa and the 10–30 kDa molecular weight range in conditioned medium from cells treated with selective Na_v_1.7 blockers are responsible for enhancing anabolism and inhibiting catabolism, respectively.

**Fig. 3:**
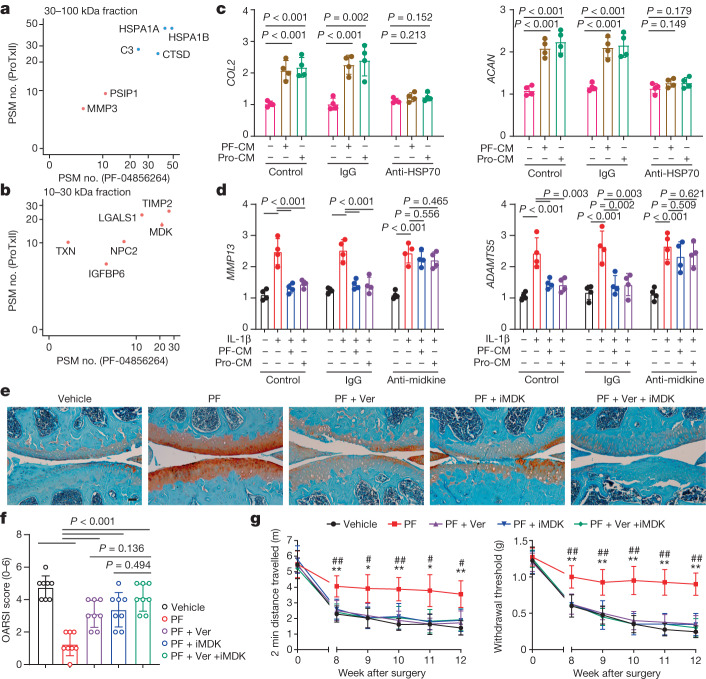
Blockade of Na_v_1.7 regulates chondrocyte biology through enhancing HSP70 and midkine secretion. **a**, Peptide-spectrum match (PSM)-based abundance of proteins identified from the 30–100 kDa fraction of conditioned medium that are unique to (red dots) or increased with (blue dots) PF-04856264 and ProTx II treatment. **b**, PSM-based abundance of proteins (red dot) identified from the 10–30 kDa fraction of conditioned medium that are unique to PF-04856264 and ProTx II treatment. **c**, *COL2* and *ACAN* mRNA levels in human C28I2 chondrocytes stimulated with conditioned medium collected from cells treated with ProTx II (Pro-CM) or PF-04856264 (PF-CM) in the absence or presence of IgG or anti-HSP70 antibodies (*n* = 4 biological replicates). **d**, *MMP13* and *ADAMTS5* mRNA levels in C28I2 chondrocytes stimulated with IL-1β and conditioned medium collected from cells treated with ProTx II (Pro-CM) or PF-04856264 (PF-CM) in the absence or presence of control IgG or anti-midkine antibodies (*n* = 4 biological replicates). **e**, Safranin O and Fast Green-stained knee joint sections (*n* = 8). Ver, VER 155008. Scale bar, 50 µm. **f**, OARSI score from images represented in **e**. **g**, Two-minute travel distance and von Frey testing at the indicated time points with indicated treatments after DMM surgery (*n* = 8). Data are mean ± 95% confidence interval, *P* values by two-way ANOVA with Bonferroni post hoc test. *Vehicle versus PF, *P* < 0.05; **vehicle versus PF, *P* *<* 0.01; ^#^PF versus PF + Ver + iMDK, *P* < 0.05; ^##^PF versus PF + Ver + iMDK, *P* < 0.01. **c**,**d**,**f**, Data are mean ± s.d., *P* values by one way ANOVA with Bonferroni post hoc test. Source Data

**Extended Data Fig. 11:**
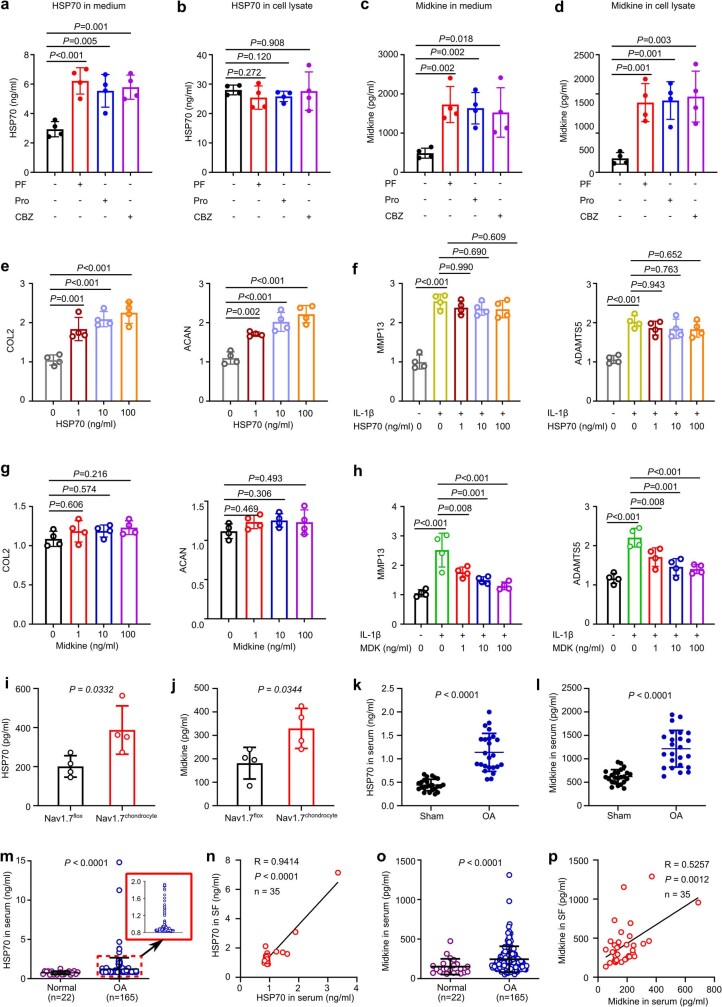
HSP70 enhances anabolism and midkine inhibits IL-1β induced catabolism in human chondrocytes. **a**, **b**, ELISA quantification of HSP70 in conditioned medium (**a**) and cell lysate (**b**) of human C28I2 cells treated with 25 nM ProTx II (Pro), 1 µM PF-04856264 (PF), or 10 µM CBZ for 48 h. **c**, **d**, ELISA quantification of Midkine in conditioned medium (**c**) and cell lysate (**d**) of human C28I2 cells treated with 25 nM Pro or 1 µM PF for 48 h. **e**, mRNA levels of *COL2* and *ACAN* in human C28I2 cells treated with serial doses of HSP70 for 24 h. **f**, mRNA levels of *MMP13* and *ADAMTS5* in human C28I2 cells treated with serial doses of HSP70 and 10 ng/ml IL-1β for 24 h. **g**, mRNA levels of *COL2* and *ACAN* in human C28I2 cells treated with serial doses of midkine for 24 h. **h**, mRNA levels of *MMP13* and *ADAMTS5* in human C28I2 cells treated with serial doses of midkine and 10 ng/ml IL-1β for 24 h. n = 4 biological replicates; Data are mean ± SD, *P* values are calculated by. **i**, **j**, ELISA quantification of HSP70 (**i**) and midkine (**j**) in conditioned medium of primary chondrocytes isolated from Nav1.7^flox^ and Nav1.7^chondrocye^ mice at 12 weeks post DMM surgery (n = 4 biological replicates). **k**, **l**, Levels of HSP70 (**k**) and midkine (**l**) in mouse sera collected from sham surgery control and from DMM surgery WT mice at 12 weeks after surgery (n = 24), assayed by ELISA. **m**, **o**, Levels of HSP70 (**m**) and midkine (**o**) in human sera collected from healthy individuals (n = 22) and from patients with OA (n = 165), assayed by ELISA. **n**, **p**, Correlation analysis (Pearson R and two-tailed P value) of HSP70 (**n**) and midkine (**p**) between matched sera and synovium fluids isolated from OA patients (n = 35). Data are means ± SD, *P* values are calculated by one way ANOVA with Bonferroni post-hoc test (**a**-**h**) and two-tailed unpaired Student’s t-test **(i**-**l**, **m**, **o**).

We analysed these two fractions of conditioned medium using tandem mass spectrometry (MS/MS). The MS/MS spectra were searched against the UniProt database, using Sequest within Proteome Discoverer. We defined candidate targets on the basis of the following properties: (1) having a molecular weight between 10 and 30 kDa or between 30 and 100 kDa; (2) exhibiting unique or more than twofold increased expression after treatment with both PF-04856264 and ProTx II relative to vehicle treatment; and (3) being secreted proteins. Of the six hits that met these criteria in the 10–30 kDa and 30–100 kDa fractions of both PF-04856264- and ProTx II-treated conditioned medium (Fig. 3a,b), we focused on HSP70 (encoded by *HSPA1A* and *HSPA1B*) and midkine (encoded by *MDK*) because these two proteins were previously reported to be implicated in chondrocyte biology, and over-expression of *HSP70* (ref. ^35^) and treatment with recombinant midkine^36^ have been reported to protect against OA. After treatment with selective Na_v_1.7 blockers and the pan-Na_v_ blocker CBZ, which is known to act on Na_v_1.7 (ref. ^26^), both HSP70 and midkine were significantly upregulated in the medium, even though the amount of HSP70 in the cell lysate remained unchanged. Midkine was also upregulated in the cell lysate (Extended Data Fig. 11a–d), suggesting that Na_v_1.7 blockers increase medium HSP70 and midkine by modulating the chondrocyte secretion. To further validate the biological regulation of HSP70 and midkine, we treated chondrocytes with the recombinant proteins. HSP70 dose-dependently enhanced chondrocyte anabolism without affecting catabolism (Extended Data Fig. 11e,f), whereas midkine dose-dependently inhibited IL-1β-induced chondrocyte catabolism without having an effect on anabolism (Extended Data Fig. 11g,h). Notably, specific blockade of HSP70 and midkine in conditioned medium using antibodies abolished the effects of conditioned medium on chondrocyte biology (Fig. 3c,d).

Given the finding that Na_v_1.7 blockers regulate chondrocyte biology by increasing secretion of HSP70 and midkine in vitro, we tested whether blocking HSP70 and/or midkine affected protective effects against OA mediated by Na_v_1.7 inhibition in vivo. Administration of the Na_v_1.7 blocker PF-04856264 resulted in a reduction in cartilage loss and a decrease of OA-associated pain behaviour in DMM mice (Fig. 3e–g). However, this protective effect was nearly abolished by combined application of the HSP70 inhibitor VER 155008 and the midkine inhibitor iMDK (Fig. 3e–g). Of note, VER 155008 or iMDK alone also significantly reduced the protective effects of PF-04856264 against OA without significantly affecting OA-associated pain (Fig. 3e–g). Collectively, these findings underscore the importance of HSP70 and midkine in maintaining the protective effects of Na_v_1.7 inhibition against OA in vivo.

Consistent with our observations that Na_v_1.7 blockers upregulate medium HSP70 and midkine in human chondrocytes, we found that genetic deletion of Na_v_1.7 increased the secretion of HSP70 and midkine in mouse chondrocytes isolated from DMM mice (Extended Data Fig. 11i,j). In addition, serum levels of HSP70 and midkine were markedly increased in DMM mice compared with sham surgery mice (Extended Data Fig. 11k,l). To verify the clinical relevance of our findings, we measured the levels of HSP70 and midkine in serum and synovial fluid from healthy individuals and patients with symptomatic knee OA (Supplementary Tables 2 and 3). Serum HSP70 and midkine levels were significantly higher in patients with OA than in healthy controls (Extended Data Fig. 11m,o). For a subset of patients with symptomatic knee OA for whom both serum and synovial fluid samples were available, serum levels of HSP70 and midkine positively correlated with synovial fluid levels (Extended Data Fig. 11n,p). Collectively, these results reveal that Na_v_1.7 blockade regulates chondrocyte anabolism and catabolism at least in part by regulating the secretions of HSP70 and midkine, respectively.

## Na_v_1.7 blockade alters chondrocyte Ca^2+^

**Fig. 4:**
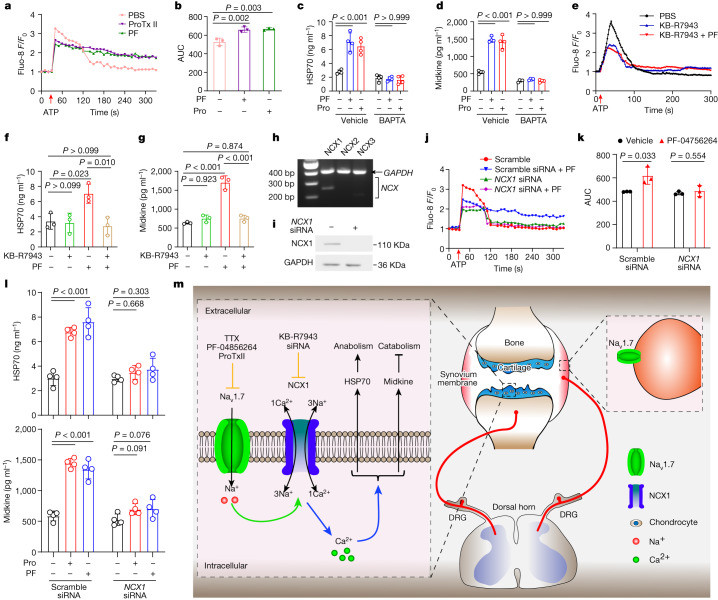
Ca^2+^ signalling in chondrocytes. **a**,**b**, *F*/*F*_0_ (**a**) and area under the curve (AUC) of intracellular Ca^2+^ (**b**) in human OA chondrocytes following ATP stimulation, measured by plate reader. ATP present from red arrow. **c**,**d**, HSP70 (**c**) and midkine (**d**) levels in conditioned medium of C28I2 cells pre-treated with BAPTA-AM, followed by ProTx II or PF-04856264. **e**, *F*/*F*_0_ of intracellular Ca^2+^ in KB-R7943 treated C28I2 chondrocytes following ATP stimulation, assayed by confocal fluorescence microscopy. **f**,**g**, HSP70 (**f**) and midkine (**g**) levels in conditioned medium of C28I2 cells treated with KB-R7943 in the presence or absence of PF-04856264. **h**, Expression of NCX isoforms in C28I2 cells. **i**, Knockdown efficiency of NCX1 in C28I2 cells. **j**,**k**, *F*/*F*_0_ (**j**) and AUC of intracellular Ca^2+^ (**k**) following ATP stimulation in C28I2 chondrocytes transfected with scramble or *NCX1* siRNA measured by plate reader. **l**, HSP70 and midkine levels in conditioned medium of chondrocytes transfected with scramble or *NCX1* siRNA and treated with ProTx II or PF-04856264. **m**, Model of mechanisms of chondrocyte- and DRG-expressed Na_v_1.7 in OA, and amelioration of OA and pain via Na_v_1.7 blockade. **b–d**,**f**,**g**,**k**,**l**, Data are mean ± s.d. **b**,**f**,**g**, *P* values calculated by one way ANOVA with Bonferroni post hoc test. **k**, Two-tailed unpaired Student’s *t*-test. **c**,**d**,**l**, Two-way ANOVA with Bonferroni post hoc test. **b**,**f**–**i**,**k**, *n* = 3 biological replicates. **c**,**d**,**l**, *n* = 4 biological replicates. Source Data

**Extended Data Fig. 12:**
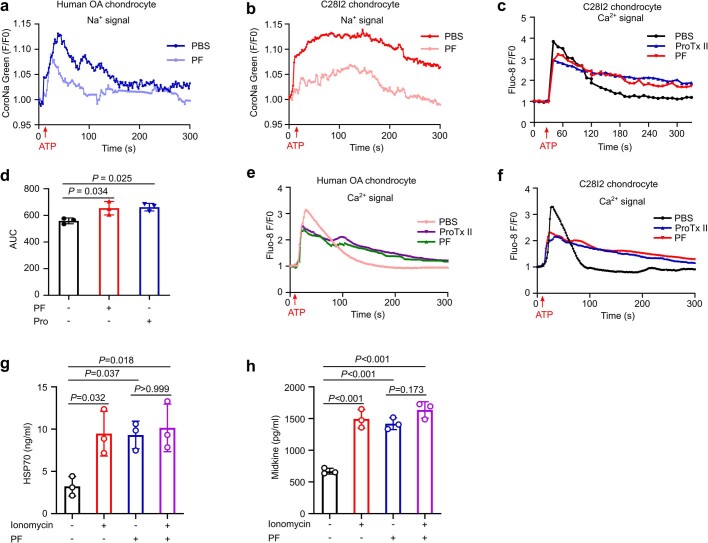
Intracellular Ca^2+^ signals are essential for the enhanced secretion of HSP70 and midkine following Nav1.7 blockade. **a**, **b**, Traces (F/F0) of intracellular Na^+^ levels over time in human OA chondrocytes (**a**) and C28I2 cells (**b**) following ATP stimulation, measured by confocal microscopy. ATP present from red arrows. **c**, **d**, Traces (F/F0) (c) and quantification of AUC (d) of intracellular Ca^2+^ levels in C28I2 chondrocytes following ATP stimulation, measured by plate reader (n = 3 biological replicates). **e**, **f**, Traces (F/F0) of intracellular Ca^2+^ levels in human OA (**e**) and C28I2 (**f**) chondrocytes following ATP stimulation, assayed by confocal fluorescence microscopy. ATP present from red arrows. **g**, **h**, ELISA quantification of HSP70 (**g**) and midkine (**h**) in conditioned medium of human C28I2 cells treated with or without Ionomycin in the absence or presence of 1 µM PF-04856264 (PF) for 48 h. n = 3 biological replicates; Data are mean ± SD, *P* values are calculated by one way ANOVA with Bonferroni post-hoc test in d, g and h.

Previous studies have shown that VGSCs contribute to the regulation of intracellular Ca^2+^ signalling in non-excitable glial cells^37^. Ca^2+^ serves as a crucial second messenger, with a pivotal role in dynamic regulation of diverse cellular processes, including protein secretion^38^. Here we investigated the role of Na_v_1.7 blockade-mediated intracellular Ca^2+^ signalling in the regulation of HSP70 and midkine secretion in chondrocytes. Initially, we assessed the effect of Na_v_1.7 inhibition on the ATP-triggered increase in intracellular Na^+^ in human OA chondrocytes and C28I2 chondrocytes. Inhibition of Na_v_1.7 with PF-04856264 attenuated the increase in intracellular Na^+^ (Extended Data Fig. 12a,b). Inhibition of Na_v_1.7 with ProTx II or PF-04856264 was followed by an increase in intracellular Ca^2+^ levels in both human OA chondrocytes and C28I2 cells. Specifically, Na_v_1.7 blockade decreased the initial intracellular Ca^2+^ surge triggered by ATP stimulation within approximately 100 s. Subsequently, Na_v_1.7 blockade resulted in higher sustained Ca^2+^ levels than in control cells. These observations were confirmed using confocal microscopy and plate reader measurements (Fig. 4a,b and Extended Data Fig. 12c–f). To validate the importance of intracellular Ca^2+^ levels in the modulation of HSP70 and midkine secretion in chondrocytes, we conducted experiments on C28I2 cells using the Ca^2+^ ionophore ionomycin and the cell-permeant Ca^2+^ chelator BAPTA-AM. The results demonstrated that higher Ca^2+^ signals contribute to the regulation of HSP70 and midkine secretion via Na_v_1.7 blockade. Notably, there was a loss of the enhanced secretion of HSP70 and midkine by Na_v_1.7 blockade in chondrocytes when BAPTA-AM was present (Fig. 4c,d and Extended Data Fig. 12g,h). We previously documented the involvement of Na^+^/Ca^2+^ exchange in the modulation of Ca^2+^ signals by VGSC blockade in astrocytes and microglial cells^37,39^. To assess the role of NCX family proteins (also known as solute carrier family 8 proteins) in regulating intracellular calcium levels after Na_v_1.7 blockade in chondrocytes, we used the pharmacological inhibitor KB-R7943 to inhibit NCX prior to ATP stimulation. These experiments showed that KB-R7943 significantly decreased the ATP-induced surge of intracellular Ca^2+^ within the first 100 s. Additionally, when NCX was blocked with KB-R7943, PF-04856264 had minimal effect on Ca^2+^ levels in chondrocytes. These results indicate that NCX contributes to the regulation of intracellular Ca^2+^ signals by Na_v_1.7 blockade (Fig. 4e). Consistent with these results, NCX inhibition abolished the enhanced secretion of HSP70 and midkine caused by Na_v_1.7 blockade (Fig. 4f,g). We also used RT–PCR to examine the expression of *NCX1* (also known as *SLC8A1*), *NCX2* (*SLC8A2*) and NCX3 (*SLC8A3*) in C28I2 cells. This experiment demonstrated clear expression of *NCX1* mRNA in the chondrocytes, whereas *NCX2* and *NCX3* were undetectable (Fig. 4h). We then showed that PF-04856264-mediated regulation of Ca^2+^ signals and secretion of HSP70 and midkine were essentially lost in chondrocytes following short interfering RNA (siRNA) knockdown of NCX1 (Fig. 4i–l). Collectively, these findings indicate that intracellular Ca^2+^ signals are essential for the enhanced secretion of HSP70 and midkine following Na_v_1.7 blockade. Moreover, these findings underscore the critical role of NCX1 in regulating calcium signalling and the associated protein secretion by Na_v_1.7 blockade in chondrocytes.

## Discussion

Although classically considered as the substrate for action potential initiation and propagation^40^, low densities of VGSCs have been reported in multiple cell types that have traditionally been considered as non-excitable, including macrophages, microglia and astrocytes, where they may contribute to the regulation of effector functions such as phagocytosis, motility, cytokine release and response to injury^41–43^. Chondrocytes express a variety of ion channel types^44,45^ that participate in diverse physiological processes, including setting resting potential, mechanoresponsiveness, volume regulation, calcium signalling, bone development, intracellular pH regulation, cellular biosynthesis and proliferation^46–51^. Notably, the expression of several ion channel types is known to be altered in OA chondrocytes^44,45,52^, whereas cartilage-specific knockout of mechanosensory ion channels decreases age-related OA^53^.

Sugimoto et al. reported a TTX-S current in rabbit articular chondrocytes^2^; however, its molecular identity was unknown. Here we demonstrate that human OA chondrocytes express TTX-S sodium current that is produced mainly by functional Na_v_1.7. We observed a sodium current density of 2.4 pA pF^−1^ (39.7 pS pF^−1^) in human chondrocytes. Assuming single-channel conductance^54^ of 6.4 pS and open probability^10,54^ of 0.4–0.6 at 0 mV, this suggests a channel density of 0.1–0.15 channels per µm^2^ and 350–525 channels per cell. Notably, TTX-S currents in human OA chondrocytes comprise 62% of the total sodium current on average, whereas ProTx II-S currents contribute 40%. This result highlights the existence of Na_v_1.7 sodium channels in primary human OA chondrocytes and points to the possibility of a fractional presence of non-Na_v_1.7 TTX-S sodium channels in this cell type.

Na_v_1.7 is known to regulate cytokine secretion in dendritic cells^42^. Here we have demonstrated that Na_v_1.7 blockers affect chondrocyte anabolic and catabolic processes through regulation of the chondrocyte secretome. The combination of fractionation of conditioned medium and subsequent proteomics analysis led to the identification of HSP70 and midkine as key molecules in conditioned medium from cells treated with Na_v_1.7 inhibitors that participate in the control of chondrocyte metabolism. Increased release of HSP70 and midkine by blockade of Na_v_1.7 in Na_v_1.7-expressing chondrocytes may have both autocrine and paracrine effects and enable Na_v_1.7-expressing and non-expressing chondrocytes to integrate signalling from the local environment to orchestrate the anabolic and catabolic processes that contribute to OA. We have demonstrated that increased intracellular Ca^2+^ signals are essential for the enhanced secretion of HSP70 and midkine induced by Na_v_1.7 blockade in chondrocytes. This effect was nullified by pharmacological inhibition and genetic ablation of NCX1, highlighting the crucial role of NCX1 in regulating intracellular Ca^2+^ signalling and subsequent secretion of HSP70 and midkine in response to the Na_v_1.7 blockade. Of note, Na_v_1.7 blockade elicits a distinct sequence of intracellular calcium level changes in chondrocytes: it initially reduces Ca^2+^ levels stimulated by ATP, which is counteracted by NCX1 inhibition; subsequently, there is a sustained elevation of Ca^2+^ levels, potentially orchestrated by a series of protein–protein and protein–lipid interactions. Our results suggest that Na_v_1.7 blockers hold promise as therapeutic agents to both protect against cartilage loss and attenuate pain in OA. We demonstrate in multiple animal models that CBZ, a sodium channel blocker currently in clinical use, prevents cartilage loss in animal models of OA, an effect beyond purely blocking pain perception. These results highlight the potential clinical application of a currently available, FDA-approved sodium channel blocker that might be repurposed for the treatment of OA.

In conclusion, we identify Na_v_1.7 as an OA-associated ion channel with dual roles in pain and cartilage homeostasis, with DRG neuron-expressed Na_v_1.7 being involved in pain and chondrocyte-expressed Na_v_1.7 governing chondrocyte biology, cartilage loss and resultant pain in OA through a powerful effect on the chondrocyte secretome (Fig. 4m). Identification of Na_v_1.7 as a novel chondrocyte-expressed, OA-associated gene uncovers a target for the development of therapies that may provide both disease-modifying and non-addictive pain relief treatment for OA.

## Methods

### Mice

C57BL/6 and *Agc1-cre*^*ERT2*^ mice were obtained from The Jackson Laboratory. *Nav1.8-cre;Nav1.7*^flox/flox^ mice were mated with transgenic mice expressing *Agc1-cre*^*ERT2*^ to obtain inducible Na_v_1.7-knockout mice in chondrocytes and both chondrocytes and DRGs. For activation of *cre*^*ERT2*^ in adult mice, 150 mg kg^−1^ body weight of tamoxifen (Sigma, T5648) in sunflower seed oil (Sigma, S5007) was injected intraperitoneally into 10-week-old mice once a day for 5 consecutive days. Littermate controls were used for all experiments. All animals were housed on a 12-h light:dark cycle with ad libitum access to food and water in a specific pathogen-free environment. Animals were maintained on a C57BL/6 J background, and age matched males typically at 12 weeks of age were used, unless otherwise specified in the figure legends. No statistical methods were used to predetermine sample size. All animal studies were performed in accordance with institutional guidelines and approved by the Institutional Animal Care and Use Committee of New York University Grossman School of Medicine.

To establish the surgically induced DMM^55^ model, after ketamine and xylazine anaesthesia, the medial meniscotibial ligament in the right knee was sectioned with a blade to destabilize the medial meniscus. The chemically induced MIA OA model was established unilaterally via intra-articular injection of 0.1 mg of MIA (Sigma, I2513) in 6 µl of 0.9% sterile saline with a 30-gauge needle after anaesthetization with ketamine and xylazine^56^. Mice with OA models were randomized to receive different treatment within a cage. PF-04856264 (Alomone labs, 1235397-05-3), at 30 µg per g body weight or 1.5 µg per g body weight was orally delivered or injected intra-articularly, respectively, daily over a 4-week period starting from the first day of MIA injection. PF-04856264 was intra-articular injected at 1.5 µg per g body weight or orally delivered at 30 µg per g body weight every other day starting from 4 weeks post DMM surgery for a total of 8 weeks. CBZ (Sigma, C4024) was delivered through oral gavage at 250 mg per kg body weight daily over a 4-week period starting from the first day of MIA injection. Additionally, doses of 10, 50 or 250 mg per kg body weight CBZ were administered daily via oral gavage over an 8-week span, commencing 4 weeks after DMM surgery. To determine whether blocking HSP70 and/or midkine affected Na_v_1.7 blocker PF-04856264’s protective effects against OA in vivo, mice received oral administration of PF-04856264 at a dosage of 30 µg per g body weight every day, and they were simultaneously subjected to intra-articular injections of VER 155008 (Sigma, SML0271) at 0.55 µg/g body weight, iMDK (Tocris, 5126) at 0.9 µg per g, or a combined application of VER 155008 and iMDK daily starting from 4 weeks after DMM surgery for a total of 8 weeks.

### Behaviour tests

OA-associated pain was measured using the von Frey assay and the open field travel analysis^57^ three times before establishment of the OA model, and every week starting from 8 weeks after DMM surgery, or at day 2, 4, 8, 14, 20 and 28 post MIA injection. All the behavioural tests were conducted in a blinded manner and performed between the hours of 12:00 and 17:00. von Frey filaments (Stoelting) were applied with increasing force intensities on the plantar surface of the hindpaw of the mouse which is placed in an elevated Plexiglass chamber with a metal grid floor that gave access to the plantar surface of the paws to determine the tactile pain threshold as based on a previous publication^57^. Rapid withdrawal of the hindpaw was recorded as a positive response. Hind paws were subjected to 10 trials at a given intensity with a 30-s interval maintained between trials and the number of positive responses for each von Frey filament’s stimulus was recorded. Animals were considered to have reached tactile threshold when 5 out of 10 trials generated a positive response. For open field travel analysis, mice were placed individually in a square clear chamber (45 × 45 cm) and allowed to freely explore for 2 min under normal lighting. Movement and trajectories of the mice were videoed and analysed by a computerized system.

### Human subjects research

Human subjects research was performed according to the Institutional Review Boards at New York University Medical Center (institutional review board (IRB) study number i11-01488 and i9018). Human OA cartilage samples were collected from patients receiving total knee joint replacement surgery for OA at New York University Langone Orthopaedic Hospital. Non-arthritic femoral condyle cartilage specimens were obtained from fresh osteochondral allografts discarded following donor plug collection during surgical osteochondral allograft implantation. Cartilage samples used are surgical discards, and no consent is required based on our approved IRB, as we do not collect patient information except age, sex and clinical diagnosis of the samples, such as Osteoarthritis. OA and non-arthritic cartilage specimens were stored in liquid nitrogen immediately after collection until protein or RNA extraction.

A total of 22 non-OA and 165 patients with symptomatic knee OA from the New York biomarker cohort^58^ were included in this study according to the American college of rheumatology (ACR) criteria. The demographic data are summarized in Supplementary Table 2. Informed consent was obtained from all participating subjects. The IRB of the New York University Grossman School of Medicine approved this study (no. i05-131).

OA synovial fluid and serum samples were collected as part of an observational study to determine factors influencing knee OA pain improvement with hyaluronic acid visco-supplementation^59^. The synovial fluid samples were collected without joint lavage, and the volume ranged from 0.5 to 30 ml. The cell-free synovial fluids were prepared and frozen (−80 °C) within 1 h of collection. Collection and storage of synovial fluids were approved (no. 13-01257) by the IRB of the NYU Grossman School of Medicine. The demographic data are summarized in Supplementary Table 3.

For the full-thickness cartilage explant assay, human tibia plateaus were obtained from 8 deidentified patients with OA undergoing total knee arthroplasty. For each individual patient with OA, 12 full-thickness cartilage explants were isolated from areas with various degrees of OA-related cartilage degeneration with a 3-mm biopsy punch and randomly distributed into three different groups and treated with 10 ng ml^−1^ IL-1β, 10 ng ml^−1^ IL-1β plus 25 nM ProTx II, or 10 ng ml^−1^ IL-1β plus 1 µM PF-04856264 in DMEM medium for 5 days. The supernatant was collected and spun at 200*g* at 4 °C, followed by ELISA assay.

### Electrophysiology

Primary human chondrocytes from patients with OA were grown in 100 mm tissue culture dishes in growth medium DMEM (Gibco, 11995-065) supplemented with 10% FBS (Hyclone, SH30088.03) and 1× penicillin–streptomycin (Thermo Fisher, 15070063). Chondrocytes were passaged every 5–7 days at 75–80% confluency no more than 3 times. Cells were plated at passaging into 12-mm round glass poly-d-lysine/laminin-coated coverslips (Corning, 354087) in 24-well plate format according to the following protocol: growth medium was removed, chondrocytes were rinsed once with 5 ml Ca^2+^ and Mg^2+^-free DPBS (Gibco, 14190-144) and incubated for 3–5 min with 1.5 ml 0.25% Trypsin/EDTA (Corning, 10222017), then cells were gently lifted off the dish and pipette-triturated in 8.5 ml of growth medium. Twenty-five microlitres of homogenized chondrocytes suspension was diluted into 1 ml growth medium at each cover glass to reach optimal cell density and were maintained in growth medium for 3–6 days until electrophysiological recordings.

Currents were recorded in whole-cell voltage clamp by Axopatch 200B amplifier (Molecular Devices). Recordings were low-pass filtered at 2 kHz and acquired at 100 kHz by Digidata 1440 A DAC using Clampex 10.7 software (Molecular Devices). p/4 leak subtraction protocol and sweep-averaging were used to subtract uncompensated leak and capacitance currents and to enhance signal/noise ratio. Pipettes were pulled from glass capillaries (PG52165-4; WPI) and had resistance 2–3.5 MΩ when filled with intracellular solution (in mM): 140 CsF, 10 NaCl, 10 HEPES, 1 EGTA, 20 dextrose, pH 7.3 with CsOH (328 mOsm l^−1^ with sucrose). Extracellular solution contained (in mM): 145 NaCl, 4 KCl, 2 CaCl_2_, 2 MgCl_2_, 10 HEPES; 10 TEA-Cl, 10 Dextrose, pH 7.4 with NaOH (327 mOsm l^−1^). Solutions for sodium current isolation were from ref. ^10^. The liquid junction potential was not compensated. Recordings were made at room temperature. Data were analysed using pClamp 10.7 (Molecular Devices) and Origin 2022b (OriginLab) software.

### RNA extraction from human cartilage

For RNA isolation, about 1 g of cartilage was pulverized in liquid nitrogen and homogenized in Trizol at a concentration of 1 g tissue per 10 ml Trizol (Invitrogen, 15596026), followed by incubation at 4 °C with rotating for 2 h. Samples were mixed with 0.2 volumes of chloroform, vortexed for 20 s, and centrifuged at 14,000 rpm for 20 min at 4 °C. The aqueous phase was collected and gently mixed with an equal volume of isopropanol, followed by centrifugation at 14,000 rpm for 20 min at 4 °C. The resulting pellet was suspended in 350 µl of RLT buffer and processed for cleanup using the RNeasy Mini Kit (Qiagen, 74104) following the manufacturer’s instructions.

### RNA assay by RT–qPCR

Total RNA extracted from chondrocytes or human cartilage was reverse transcribed using the High-Capacity cDNA Reverse Transcription Kit (Applied Biosystems, 4387406). RT–qPCR was performed in triplicate with SYGR Green (Applied Biosystems, A25780) using human or mouse primers to *Acan*, *Col2*, *Mmp13*, *Adamts5*, *Cox2*, *Nos2* and *Gapdh* (Applied Biosystems Real-time PCR system). mRNA levels were normalized to *Gapdh* and reported as relative mRNA fold change.

### Histology

Human cartilage or mouse knee joints were fixed in 4% paraformaldehyde for 24 h before decalcification in 10% w/v EDTA for 2 weeks before paraffin embedding. The paraffin blocks were sectioned at a thickness of 5 µm and serial sections were subjected to Safranin O or haematoxylin and eosin (H&E) staining. Cartilage destruction was graded on Safranin O-stained sections by blinded observers using the OARSI histology scoring system^60^ (grade 0–6). Osteophyte development^61^ (grade 0–3) was evaluated and the thickness of the subchondral bone plate^62^ was measured on Safranin O or H&E-stained sections. Synovitis (grade 0–9) was determined based on the synovial lining cell layer enlargement, resident cell density, and inflammatory infiltration on H&E-stained sections^63^. For immunohistochemical staining, deparaffinized and hydrated sections were incubated with 0.1% trypsin for 30 min at 37 °C, followed by 0.25 U ml^−1^ chondroitinase ABC (Sigma-Aldrich, C3667) and 1 U ml^−1^ hyaluronidase (Sigma-Aldrich, H3560) for 60 min at 37 °C, respectively. After blocking, the sections were incubated with antibodies against Na_v_1.7 (1:50, Alomone Labs, ASC-008), COL2 (Invitrogen, cat. no. MA5-12789), COMP fragment^64^ (1:200, affinity-purified monoclonal), aggrecan neoepitope (1:100, Millipore, AB8135) and MMP13 (1:200, Abcam, ab3208) overnight at 4 °C. Detection was performed using the Vectastain Elite ABC kit (Vector Laboratories, PK6100), and the positive signal was visualized with 0.5 mg ml^−1^ 3,3-diaminobenzidine in 50 mM Tris-Cl substrate (Sigma-Aldrich, D12384) and then counterstained with 1% methyl green (Sigma-Aldrich, 67060). Images were acquired with a Zeiss microscope. Semi-quantification analysis of the density of immunohistochemical staining for Aggrecan neoepitope, COMP fragment and collagen X was performed by ImageJ, and the same signal threshold was used for each group of similar immunohistochemical images^65^.

### Cell culture

Primary articular chondrocytes were isolated from the femoral condyles and tibial plateaus of *Nav1.7*^*flox*^ and *Nav1.7*^*chondrocyte*^ mice on postnatal day 6 (ref. ^66^). Chondrocytes were maintained as a monolayer in Dulbecco’s modified Eagle’s medium (DMEM) supplemented with 10% FBS, 50 U/ml penicillin, and 0.05 mg ml^−1^ streptomycin. Articular chondrocytes at culture day 2 were treated as indicated for each experiment.

Primary articular chondrocytes were isolated from the femoral condyles and tibial plateaus of *Nav1.7*^*flox*^ and *Nav1.7*^*chondrocyte*^ mice at 12 weeks after DMM surgery. Chondrocytes were maintained as a monolayer in Dulbecco’s modified Eagle’s medium (DMEM) supplemented with 10% FBS, 50 U ml^−1^ penicillin, and 0.05 mg ml^−1^ streptomycin. After 5 days, the conditioned medium was collected and spun at 200*g* at 4 °C, followed by ELISA assay.

Human C28I2 chondrocytes were grown in DMEM medium supplemented with 10% FBS, 50 U ml^−1^ penicillin, and 0.05 mg ml^−1^ streptomycin. To knockdown Na_v_1.7, cells were transfected with commercially available siRNA (S534077, A134907) using Lipofectamine (Invitrogen, 13778100) as instructed by manufacture’s protocol. To fraction condition medium, C28I2 cells were stimulated with or without 25 nM ProTx II or PF-04856264. In brief, C28I2 cells were cultured with DMEM medium supplemented with 10% FBS, 50 U ml^−1^ penicillin and 0.05 mg ml^−1^ streptomycin. When cells reached 80% confluency, the medium was changed to DMEM supplemented with ITS Liquid Media Supplement (Sigma-Aldrich, I3146) containing 25 nM ProTx II or PF-04856264 for 2 days. The conditioned medium was then collected. After centrifugation to get rid of the cell debris, the medium was then fractioned based on molecular weight to >100 kDa, 30–100 kDa, 10–30 kDa and <10 kDa using Amicon Ultra-2 Centrifugal Filter Units sequentially (Fisher Scientific, UFC210024, UFC203024 and UFC201024). PF-04856264 and ProTx II have molecular weight of 437.492 and 3,826.65 Da, respectively. To exclude the effects of PF-04856264 and ProTx II in conditioned medium, ProTx II was depleted using dialysis tubing that allows the removal of molecules with molecular weights between 3.5–5 kDa (Micro Float-A-Lyzer 3.5–5 kDa, F235053, Thomas Scientific), while PF-04856264 was simply removed through dialysis against the medium. Conditioned medium with molecular weight of 30–100 kDa and 10–30 kDa were then analysed by mass spectrometry, performed by NYU Proteomics Laboratory. All MS/MS spectra were collected using the following instrument parameters: resolution of 15,000, automatic gain control (AGC) target of 5e4, maximum ion time of 120 ms, one microscan, 2 *m*/*z* isolation window, fixed first mass of 150 *m*/*z*, and normalized collision energy (NCE) of 27. MS/MS spectra were searched against a UniProt Human database using Sequest within Proteome Discoverer 1.4.

### Western blotting

Western blot analyses were conducted with protein lysates from primary human cartilage and C28I2 cells. To determine the membrane localization of Na_v_1.7 in chondrocytes, cytosolic and membrane fractions of human C28I2 cells were extracted with Men-PER Plus Membrane Protein Extraction Kit (Thermo Fisher Scientific, 89842) and subjected to western blotting analysis. The following primary antibodies were used: Na_v_1.7 (1:500, Alomone Labs, ASC-008), TNFR2 (1:1,000, ProteinTech, 19272-1-AP), and GAPDH (1:5,000, ProteinTech, 60004-1-Ig).

### ELISA

The levels of HSP70 and midkine in human sera and synovial fluid from healthy individuals and patients with OA, in conditioned medium and cell lysates of human C28I2 cells treated with 25 nM ProTx II or 1 µM PF-04856264 were measured by ELISA according to the manufacture’s instructions (Abcam, ab133060, ab193761). Before ELISA analysis, human synovial fluids were digested with hyaluronidase at 1 unit per 100 µl synovial fluid for 1 h at 37 °C. The levels of PRG4 and MMP13 in supernatant of human OA cartilage explants were measured by ELISA according to the manufacturer’s instructions, respectively (R&D systems, DY9829-05; Abcam, ab100605). The levels of HSP70 and midkine in mouse sera and conditioned medium collected from primary chondrocytes were measured by ELISA according to the manufacture’s instructions (Abcam, ab133061, ab279416).

### Na^+^ and Ca^2+^ fluorescence imaging

Human OA chondrocytes or C28I2 cells were seeded on 8-well chamber (Thermo Fisher, 154461) and loaded with 5 µM CoroNa Green (Invitrogen, C36676) or Fluo-8 (Abcam, ab112129) in Hanks’ Balanced Salt Solution for 45 min at 37 °C in the presence or absence of 25 nM ProTx II or 1 µM PF-04856264 with or without 0.5 µM KB-R7943 (Tocris, 1244). CoroNa Green and Fluo-8 were excited at 488 nm and fluorescence images (525–530 nm) were acquired with 25x water-dipping objective on Zeiss 880 confocal microscope every 2 s during the experiment. Na^+^ and Ca^2+^ transients in chondrocytes were induced by 100 nM ATP in the presence or absence of 25 nM ProTx II or 1 µM PF-04856264. Fluorescence was expressed as the ratio of cytosolic fluorescence and initial intensity (*F*/*F*_0_).

### Measurement of intracellular Ca^2+^ with plate reader

Intracellular Ca^2+^ in human OA chondrocytes or C28I2 cells was measured using Fluo-8 Calcium Flux Assay Kit (Abcam, ab112129) according to the manufacturer’s instructions. In brief, cells seeded in black walled 96-well plate (Corning, 3904) were loaded with Fluo-8 in Hanks’ Balanced Salt Solution for 30 min at 37 °C and 30 min at room temperature in the presence or absence of 25 nM ProTx II or 1 µM PF-04856264. Ca^2+^ transients in chondrocytes were induced by 100 nM ATP in the presence or absence of 25 nM ProTx II or 1 µM PF-04856264. Fluorescence was measured in a fluorescent plate reader with an excitation wavelength of 490 nm and emission wavelength of 525 nm, and expressed as the ratio of cytosolic fluorescence and initial intensity (*F*/*F*_0_).

### Statistical analysis

All data are presented as mean ± s.d., unless otherwise specified in the figure or table legends. The numbers of mice used per genotype are indicated in figure legends. Comparisons between the two groups were analysed using two-tailed unpaired Student’s *t*-test unless stated otherwise in the figure legends. ANOVA with post hoc Bonferroni test was used when comparing multiple groups as described in the figure legends. A value of *P* < 0.05 was considered statistically significant. Statistical analyses were performed using GraphPad Prism 9.

### Reporting summary

Further information on research design is available in the Nature Portfolio Reporting Summary linked to this article.

## Online content

Any methods, additional references, Nature Portfolio reporting summaries, source data, extended data, supplementary information, acknowledgements, peer review information; details of author contributions and competing interests; and statements of data and code availability are available at 10.1038/s41586-023-06888-7.

### Supplementary information

Supplementary Information This file contains a supplementary discussion, Supplementary Figs. 1 and 2, Supplementary Tables 1–3 and references.

Reporting Summary

Peer Review File

### Source data

Source Data Figs. 3 and 4 and Source Data Extended Data Figs. 2–6 and 10.

## Data Availability

Full immunoblots are provided as Supplementary Fig. 1. Source data are provided with this paper.

## References

[1] Katz JN, Arant KR, Loeser RF (2021). Diagnosis and treatment of hip and knee osteoarthritis: a review. JAMA.

[2] Sugimoto T, Yoshino M, Nagao M, Ishii S, Yabu H (1996). Voltage-gated ionic channels in cultured rabbit articular chondrocytes. Comp. Biochem. Physiol. C.

[3] Loeser RF (2012). Microarray analysis reveals age-related differences in gene expression during the development of osteoarthritis in mice. Arthritis Rheum..

[4] Abramson SB, Attur M, Yazici Y (2006). Prospects for disease modification in osteoarthritis. Nat. Clin. Pract. Rheumatol..

[5] Fu K, Robbins SR, McDougall JJ (2018). Osteoarthritis: the genesis of pain. Rheumatology.

[6] Miller RE (2015). The role of peripheral nociceptive neurons in the pathophysiology of osteoarthritis pain. Curr. Osteoporos. Rep..

[7] Dib-Hajj SD, Cummins TR, Black JA, Waxman SG (2010). Sodium channels in normal and pathological pain. Annu. Rev. Neurosci..

[8] Catterall WA, Goldin AL, Waxman SG (2003). International Union of Pharmacology. XXXIX. Compendium of voltage-gated ion channels: sodium channels. Pharmacol. Rev..

[9] Zhu J (2020). Aberrant subchondral osteoblastic metabolism modifies Na_v_1.8 for osteoarthritis. eLife.

[10] Vasylyev DV, Han C, Zhao P, Dib-Hajj S, Waxman SG (2014). Dynamic-clamp analysis of wild-type human Na_v_1.7 and erythromelalgia mutant channel L858H. J. Neurophysiol..

[11] Cao L (2016). Pharmacological reversal of a pain phenotype in iPSC-derived sensory neurons and patients with inherited erythromelalgia. Sci. Transl. Med..

[12] Drenth JP, Waxman SG (2007). Mutations in sodium-channel gene SCN9A cause a spectrum of human genetic pain disorders. J. Clin. Invest..

[13] Cox JJ (2006). An SCN9A channelopathy causes congenital inability to experience pain. Nature.

[14] Bennett DL, Clark AJ, Huang J, Waxman SG, Dib-Hajj SD (2019). The role of voltage-gated sodium channels in pain signaling. Physiol. Rev..

[15] Reimann F (2010). Pain perception is altered by a nucleotide polymorphism in SCN9A. Proc. Natl Acad. Sci. USA.

[16] Nassar MA, Levato A, Stirling LC, Wood JN (2005). Neuropathic pain develops normally in mice lacking both Na_v_1.7 and Na_v_1.8. Mol. Pain.

[17] Nassar MA (2004). Nociceptor-specific gene deletion reveals a major role for Na_v_1.7 (PN1) in acute and inflammatory pain. Proc. Natl Acad. Sci. USA.

[18] Rahman W, Dickenson AH (2015). Osteoarthritis-dependent changes in antinociceptive action of Na_v_1.7 and Na_v_1.8 sodium channel blockers: An in vivo electrophysiological study in the rat. Neuroscience.

[19] Black JA, Waxman SG (2013). Noncanonical roles of voltage-gated sodium channels. Neuron.

[20] Mapp PI, Walsh DA (2012). Mechanisms and targets of angiogenesis and nerve growth in osteoarthritis. Nat. Rev. Rheumatol..

[21] Malfait AM, Miller RJ (2016). Emerging targets for the management of osteoarthritis pain. Curr. Osteoporos. Rep..

[22] Fu W (2021). 14-3-3 epsilon is an intracellular component of TNFR2 receptor complex and its activation protects against osteoarthritis. Ann. Rheum. Dis..

[23] Cai D, Yin S, Yang J, Jiang Q, Cao W (2015). Histone deacetylase inhibition activates Nrf2 and protects against osteoarthritis. Arthritis Res. Ther..

[24] Sousa-Valente J (2018). Role of TrkA signalling and mast cells in the initiation of osteoarthritis pain in the monoiodoacetate model. Osteoarthritis Cartilage.

[25] McCormack K (2013). Voltage sensor interaction site for selective small molecule inhibitors of voltage-gated sodium channels. Proc. Natl Acad. Sci. USA.

[26] Yang Y, Mis MA, Estacion M, Dib-Hajj SD, Waxman SG (2018). Na_v_1.7 as a pharmacogenomic target for pain: moving toward precision medicine. Trends Pharmacol. Sci..

[27] Nair AB, Jacob S (2016). A simple practice guide for dose conversion between animals and human. J. Basic Clin. Pharm..

[28] Forouhan M, Sonntag S, Boot-Handford RP (2018). Carbamazepine reduces disease severity in a mouse model of metaphyseal chondrodysplasia type Schmid caused by a premature stop codon (Y632X) in the *Col10a1* gene. Hum. Mol. Genet..

[29] Schmalhofer WA (2008). ProTx-II, a selective inhibitor of Na_v_1.7 sodium channels, blocks action potential propagation in nociceptors. Mol. Pharmacol..

[30] Molnar V (2021). Cytokines and chemokines involved in osteoarthritis pathogenesis. Int. J. Mol. Sci..

[31] Li C (2017). Double-stranded RNA released from damaged articular chondrocytes promotes cartilage degeneration via Toll-like receptor 3-interleukin-33 pathway. Cell Death Dis..

[32] Das N, Schmidt TA, Krawetz RJ, Dufour A (2019). Proteoglycan 4: from mere lubricant to regulator of tissue homeostasis and inflammation: does proteoglycan 4 have the ability to buffer the inflammatory response?. Bioessays.

[33] Ashcroft FM, Harrison DE, Ashcroft SJ (1984). Glucose induces closure of single potassium channels in isolated rat pancreatic β-cells. Nature.

[34] Yang SN, Berggren PO (2006). The role of voltage-gated calcium channels in pancreatic beta-cell physiology and pathophysiology. Endocr. Rev..

[35] Son YO, Kim HE, Choi WS, Chun CH, Chun JS (2019). RNA-binding protein ZFP36L1 regulates osteoarthritis by modulating members of the heat shock protein 70 family. Nat. Commun..

[36] Xu C (2014). The therapeutic effect of rhMK on osteoarthritis in mice, induced by destabilization of the medial meniscus. Biol. Pharm. Bull..

[37] Persson AK (2014). Contribution of sodium channels to lamellipodial protrusion and Rac1 and ERK1/2 activation in ATP-stimulated microglia. Glia.

[38] Anantharam A, Kreutzberger AJB (2019). Unraveling the mechanisms of calcium-dependent secretion. J. Gen. Physiol..

[39] Pappalardo LW, Samad OA, Black JA, Waxman SG (2014). Voltage-gated sodium channel Na_v_1.5 contributes to astrogliosis in an in vitro model of glial injury via reverse Na^+^/Ca^2+^ exchange. Glia.

[40] Hodgkin AL, Huxley AF (1952). A quantitative description of membrane current and its application to conduction and excitation in nerve. J. Physiol..

[41] Craner MJ (2005). Sodium channels contribute to microglia/macrophage activation and function in EAE and MS. Glia.

[42] Kis-Toth K (2011). Voltage-gated sodium channel Na_v_1.7 maintains the membrane potential and regulates the activation and chemokine-induced migration of a monocyte-derived dendritic cell subset. J. Immunol..

[43] Sontheimer H, Black JA, Waxman SG (1996). Voltage-gated Na^+^ channels in glia: properties and possible functions. Trends Neurosci..

[44] Lewis R, May H, Mobasheri A, Barrett-Jolley R (2013). Chondrocyte channel transcriptomics: do microarray data fit with expression and functional data?. Channels.

[45] Mobasheri A (2019). The chondrocyte channelome: a narrative review. Joint Bone Spine.

[46] Wilson JR, Duncan NA, Giles WR, Clark RB (2004). A voltage-dependent K^+^ current contributes to membrane potential of acutely isolated canine articular chondrocytes. J. Physiol..

[47] Maleckar MM, Clark RB, Votta B, Giles WR (2018). The resting potential and K^+^ currents in primary human articular chondrocytes. Front. Physiol..

[48] Phan MN (2009). Functional characterization of TRPV4 as an osmotically sensitive ion channel in porcine articular chondrocytes. Arthritis Rheum..

[49] Qian N (2019). TRPM7 channels mediate spontaneous Ca^2+^ fluctuations in growth plate chondrocytes that promote bone development. Sci. Signal..

[50] Savadipour A, Nims RJ, Katz DB, Guilak F (2022). Regulation of chondrocyte biosynthetic activity by dynamic hydrostatic pressure: the role of TRP channels. Connect. Tissue Res..

[51] Ponce A, Jimenez-Pena L, Tejeda-Guzman C (2012). The role of swelling-activated chloride currents (*I*_CL,swell_) in the regulatory volume decrease response of freshly dissociated rat articular chondrocytes. Cell. Physiol. Biochem..

[52] Matta C (2021). Transcriptome-based screening of ion channels and transporters in a migratory chondroprogenitor cell line isolated from late-stage osteoarthritic cartilage. J. Cell. Physiol..

[53] O’Conor CJ (2016). Cartilage-specific knockout of the mechanosensory ion channel TRPV4 decreases age-related osteoarthritis. Sci. Rep..

[54] Sigworth FJ (1980). The variance of sodium current fluctuations at the node of Ranvier. J. Physiol..

[55] Glasson SS, Blanchet TJ, Morris EA (2007). The surgical destabilization of the medial meniscus (DMM) model of osteoarthritis in the 129/SvEv mouse. Osteoarthritis Cartilage.

[56] Pitcher T, Sousa-Valente J, Malcangio M (2016). The monoiodoacetate model of osteoarthritis pain in the mouse. J. Vis. Exp..

[57] Ruan MZ, Patel RM, Dawson BC, Jiang MM, Lee BH (2013). Pain, motor and gait assessment of murine osteoarthritis in a cruciate ligament transection model. Osteoarthritis Cartilage.

[58] Attur M (2015). Plasma levels of interleukin-1 receptor antagonist (IL1Ra) predict radiographic progression of symptomatic knee osteoarthritis. Osteoarthritis Cartilage.

[59] Bournazou E (2019). Vascular adhesion protein-1 (VAP-1) as predictor of radiographic severity in symptomatic knee osteoarthritis in the New York University Cohort. Int. J. Mol. Sci..

[60] Glasson SS, Chambers MG, Van Den Berg WB, Little CB (2010). The OARSI histopathology initiative—recommendations for histological assessments of osteoarthritis in the mouse. Osteoarthritis Cartilage.

[61] Little CB (2009). Matrix metalloproteinase 13-deficient mice are resistant to osteoarthritic cartilage erosion but not chondrocyte hypertrophy or osteophyte development. Arthritis Rheum..

[62] Das Neves Borges P, Vincent TL, Marenzana M (2017). Automated assessment of bone changes in cross-sectional micro-CT studies of murine experimental osteoarthritis. PLoS ONE.

[63] Krenn V (2006). Synovitis score: discrimination between chronic low-grade and high-grade synovitis. Histopathology.

[64] Lai Y (2014). ADAMTS-7 forms a positive feedback loop with TNF-α in the pathogenesis of osteoarthritis. Ann. Rheum. Dis..

[65] Crowe AR, Yue W (2019). Semi-quantitative determination of protein expression using immunohistochemistry staining and analysis: an integrated protocol. Bio Protoc..

[66] Gosset M, Berenbaum F, Thirion S, Jacques C (2008). Primary culture and phenotyping of murine chondrocytes. Nat. Protoc..

