# Supplementary material for: Nav1.7 as a chondrocyte regulator and therapeutic target for osteoarthritis
Source: Nature. 2024 Jan 3;625(7995):557–65. doi: 10.1038/s41586-023-06888-7 (PMC10794151; doi:10.1038/s41586-023-06888-7)

nature portfolio

https://doi.org/10.1038/s41586-023-06888-7

Supplementary information

$Na_v1.7$ as a chondrocyte regulator and therapeutic target for osteoarthritis

In the format provided by the authors and unedited

Supplementary Information for

Nav1.7 as chondrocyte regulator and therapeutic target for osteoarthritis

Wenyu Fu[1,2], Dmytro Vasylyev[3,4], Yufei Bi[1], Mingshuang Zhang[1], Guodong Sun[1], Asya Khleborodova[1], Guiwu Huang[1,2], Libo Zhao[1,2], Renpeng, Zhou[1,2], Yonggang Li[1,2], Shujun Liu[3,4], Xianyi Cai[1], Wenjun He[1], Min Cui[1], Xiangli Zhao[1,2], Aubryanna Hettinghouse[1], Julia Good[1], Ellen Kim[1], Eric Strauss[1], Philipp Leucht[1], Ran Schwarzkopf[1], Edward X Guo[5], Jonathan Samuels[6], Wenhuo Hu[7], Mukundan Attur[6], Stephen G Waxman[3,4¶], Chuan-ju Liu[1,2, 8¶]

1. Department of Orthopaedic Surgery, New York University Grossman School of Medicine, New York, NY, 10003, USA
2. Department of Orthopaedics and Rehabilitation, Yale University School of Medicine, New Haven, CT, 06519, USA
3. Department of Neurology, Yale School of Medicine, New Haven, CT, 06520, USA
4. Center for Neuroscience and Regeneration Research, Veterans Affairs Connecticut Healthcare, West Haven, CT, 06515, USA
5. Department of Biomedical Engineering, Columbia University, New York, NY, 10027, USA
6. Department of Medicine, Division of Rheumatology, Department of Medicine, New York University Grossman School of Medicine, New York, NY, 10003, USA
7. Human Oncology and Pathogenesis Program, Memorial Sloan Kettering Cancer Center; Marie-Josée and Henry R. Kravis Center for Molecular Oncology, Memorial Sloan Kettering Cancer Center, New York, NY, 10065, USA
8. Department of Cell Biology, New York University Grossman School of Medicine, New York, NY, 10003, USA

[¶]To whom correspondence should be addressed: chuan-ju.liu@yale.edu; or stephen.waxman@yale.edu

This PDF file includes:

- Supplementary Discussion (Page 3-4)
- References for Supplementary Discussion (Page 5)
- Supplementary Table 1 (Expression of fast-inactivating sodium currents in human OA chondrocytes, Page 6)
- Supplementary Table 2 (Demographic data of normal and OA patients, Page 7)
- Supplementary Table 3 (Demographic data of OA patients with serum and synovial fluids, Page 8)
- Supplementary Fig. 1 (Uncropped western blot gels for Fig.4i and Extended Data Fig. 1e,f, Page 9)
- Supplementary Fig. 2 (Uncropped DNA gels for Fig. 4h and Extended Data Fig. 1a, Page 10)

Supplementary Discussion

OA is a multifactorial disease, defined by a combination of radiographic cartilage loss and chronic pain. Identification of potential genes that concurrently control OA disease progression and pain is a long-sought goal and remains a big challenge [1]. Our study pinpoints Nav1.7 as a molecule with such dual roles in OA. Data from multiple genetically-engineered mice reveal the relative contribution of chondrocyte-, and DRG neuron-expressed Nav1.7 to cartilage protection and pain relief in OA. Genetic ablation of Nav1.7 in chondrocytes concurrently attenuates cartilage loss and alleviates OA pain, while deletion of Nav1.7 in DRG neurons only reduces OA pain without disease-modifying effects in OA cartilage; and deletion of Nav1.7 in both DRG neurons and chondrocytes confers prominent protection against OA progression and OA pain. The fact that Nav1.7 deletion in DRG neurons only alleviates OA pain further emphasizes the importance of chondrocyte-expressed Nav1.7 in regulating cartilage homeostasis during the pathogenesis of OA.

OA pain is believed to arise from many sources [2]. Although there is an established link between cartilage loss and OA pain [3], the contribution of cartilage loss to OA pain is controversial given the weak correlation between radiographic evidence of OA and the main clinical presentation of OA pain [4]. Our genetic studies indicate that OA pain can be attenuated by specific deletion of Nav1.7 in chondrocytes by a mechanism that involves protection against cartilage loss, suggesting that the association of cartilage loss with pain could be pathogenically important.

Serum HSP70 and midkine levels are elevated in OA patients compared to healthy controls. This paradoxical observation aligns with evidence that OA chondrocytes also exhibit increased anabolic activity, such as increased levels of anabolic growth factors [5,6].

Our results suggest that Nav1.7 blockers hold promise as therapeutic agents that can both protect against cartilage loss in OA and attenuate OA pain. We demonstrate in multiple animal models that CBZ, a sodium channel blocker currently in clinical use, prevents cartilage loss in animal models of OA, an effect beyond purely blocking pain perception. These results highlight the potential clinical application of a currently available, FDA-approved sodium channel blocker which might be repurposed for the treatment of OA.

Genetic deletion and pharmacological blockade of Nav1.7 provide proof-of-principle that targeting of Nav1.7 can protect against cartilage destruction, ameliorate OA progression, and

attenuate OA-associated pain. These findings provide the first evidence demonstrating that chondrocytes express functional Nav1.7 channels, and demonstrate the importance of Nav1.7 in the regulation of chondrocytes and OA. Our observations also suggest a new avenue for the development of Nav1.7 blockers as novel disease-modifying drugs for treating OA pathologically and symptomatically, thereby expanding their clinical utility beyond that of relief from neuronal hyperexcitability.

References for Supplementary Discussion:

1 Thakur, M., Dawes, J. M. & McMahon, S. B. Genomics of pain in osteoarthritis. *Osteoarthritis Cartilage* **21**, 1374-1382, doi:10.1016/j.joca.2013.06.010 (2013).

2 Jones, G. Pain in OA: is cartilage loss a major contributor? *Nat Rev Rheumatol* **16**, 541-542, doi:10.1038/s41584-020-0484-3 (2020).

3 Zhai, G. *et al.* Correlates of knee pain in older adults: Tasmanian Older Adult Cohort Study. *Arthritis Rheum* **55**, 264-271, doi:10.1002/art.21835 (2006).

4 Bacon, K., LaValley, M. P., Jafarzadeh, S. R. & Felson, D. Does cartilage loss cause pain in osteoarthritis and if so, how much? *Ann Rheum Dis* **79**, 1105-1110, doi:10.1136/annrheumdis-2020-217363 (2020).

5 Guo, F. *et al.* Granulin-epithelin precursor binds directly to ADAMTS-7 and ADAMTS-12 and inhibits their degradation of cartilage oligomeric matrix protein. *Arthritis Rheum* **62**, 2023-2036, doi:10.1002/art.27491 (2010).

6 Sandell, L. J. & Aigner, T. Articular cartilage and changes in arthritis. An introduction: cell biology of osteoarthritis. *Arthritis Res* **3**, 107-113, doi:10.1186/ar148 (2001).

Supplementary Table 1. Expression of fast-inactivating sodium currents in human OA chondrocytes.

	Age (Year)	Gender	KL	I (PA)	Id (pA/pF)	Cm (pF)	#cells	%express
Patient1	73	F	4	32 (n=1)	1.5(n=1)	33.4 ± 2.6 (n=12)	12	8
Patient2	52	M	4	94 ± 14 (n=10)	2.6 ± 0.5 (n=10)	36.9 ± 1.2 (n=52)	52	19
Patient3	58	F	3	49 ± 19 (n=2)	1.8 ± 0.8 (n=2)	30.1 ± 2.2 (n=13)	13	15
Combined				82 ± 19 (n=13)	2.4 ± 0.4 (n=13)	35.2 ± 1.2 (n=77)	77	17

Shown are (means ± s.e.). I (pA), peak amplitude of total sodium current at 0 mV test voltage in chondrocytes expressing fast-kinetics sodium current; Id (pA/pF), current density; Cm (pF), membrane capacitance; #cells, number of cells recorded; % express – percentage of cells expressing fast-kinetics sodium current.

Supplementary Table 2. Demographic data of normal and OA patients

	Non-OA (n=22)	Knee OA (n=165)
Age (Year)	55.23 ± 7.96	63.13 ± 10.33
BMI	26.14 ± 3.83	27.28 ± 4.19
Gender	F (n=16), M (n=6)	F (n=105), M (n=60)
KL 0	22	
KL 1		17
KL 2		28
KL 3		74
KL 4		46

Supplementary Table 3. Demographic data of OA patients with serum and synovial fluids

	Serum /Synovial fluid (n=35)
Age (Year)	62.73 ± 11.95
BMI	29.64 ± 4.36
Gender	F (n=21), M (n=14)
KL 2	3
KL 3	22
KL 4	10

Supplementary Fig.1 Uncropped gels for Fig. 4i and Extended Data Fig.1e,f. After electrophoretic transfer of proteins, the NC membranes were cut into strips containing sets of samples, and were then subjected to immunoblotting. Proteins were detected by using the indicated antibodies and the black rectangles show the cropping location.

Fig. 4i

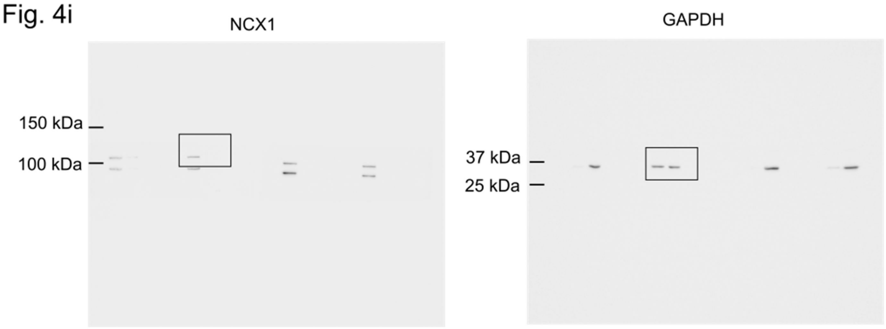

ED Fig. 1e

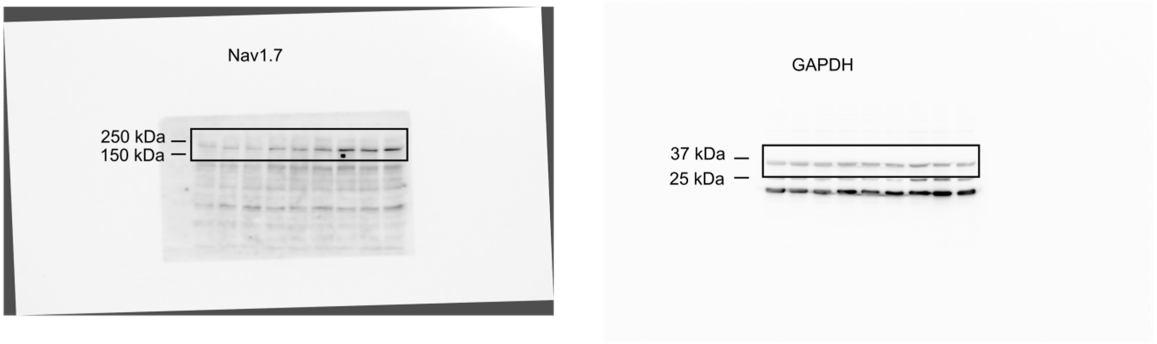

ED Fig. 1f

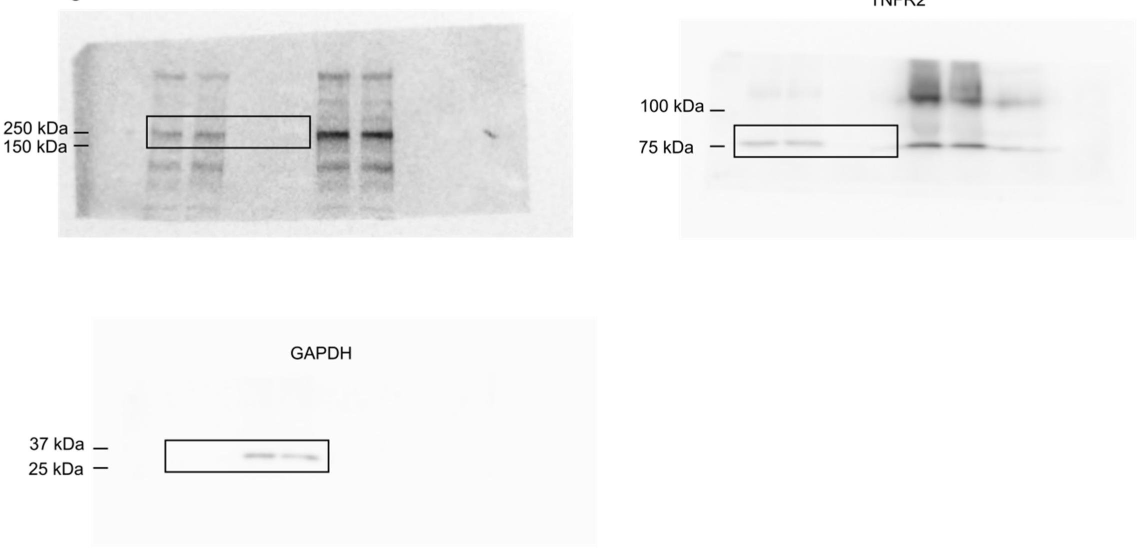

Supplementary Fig.2 Uncropped DNA gels for Fig. 4h and Extended Data Fig.1a.

Fig. 4h

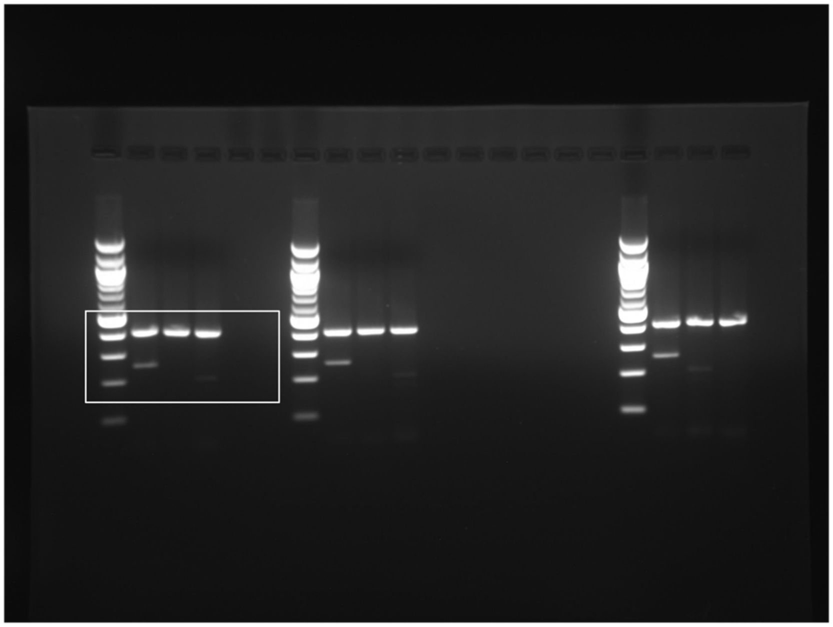

ED Fig. 1a

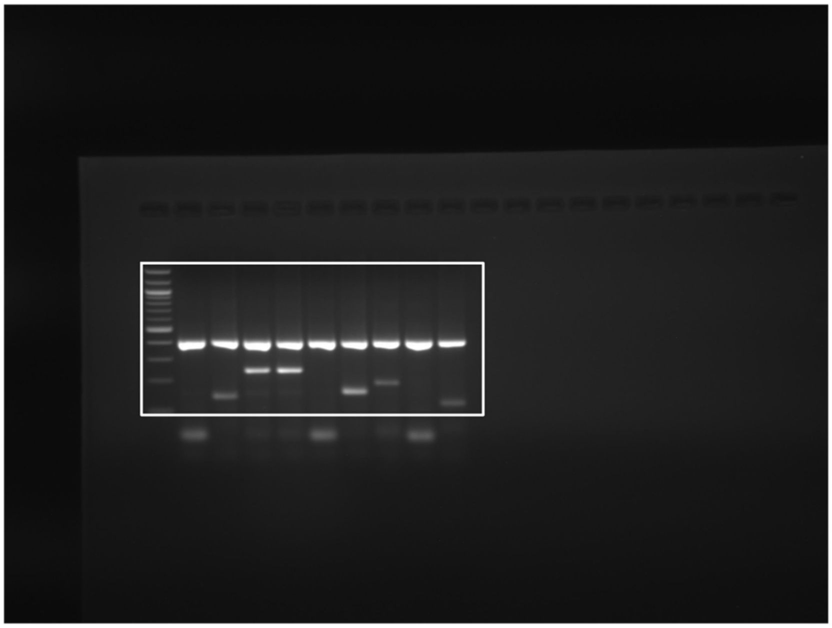

Supplement: Supplementary file 1 — This file contains a supplementary discussion, Supplementary Figs. 1 and 2, Supplementary Tables 1–3 and references. [file 41586_2023_6888_MOESM1_ESM.pdf]
