# Supplementary material for: Nav1.7 as a chondrocyte regulator and therapeutic target for osteoarthritis
Source: Nature. 2024 Jan 3;625(7995):557–65. doi: 10.1038/s41586-023-06888-7 (PMC10794151; doi:10.1038/s41586-023-06888-7)
Supplement: Supplementary file 2 — Reporting Summary [file 41586_2023_6888_MOESM2_ESM.pdf]

nature portfolio

Corresponding author(s): Stephen G. Waxman; Chuan-ju Liu

Last updated by author(s): Oct 6, 2023

Reporting Summary

Nature Portfolio wishes to improve the reproducibility of the work that we publish. This form provides structure for consistency and transparency in reporting. For further information on Nature Portfolio policies, see our Editorial Policies and the Editorial Policy Checklist.

Statistics

For all statistical analyses, confirm that the following items are present in the figure legend, table legend, main text, or Methods section.

n/a	Confirmed	
☐	☒	The exact sample size (n) for each experimental group/condition, given as a discrete number and unit of measurement
☐	☒	A statement on whether measurements were taken from distinct samples or whether the same sample was measured repeatedly
☐	☒	The statistical test(s) used AND whether they are one- or two-sided<br>*Only common tests should be described solely by name; describe more complex techniques in the Methods section.*
☒	☐	A description of all covariates tested
☐	☒	A description of any assumptions or corrections, such as tests of normality and adjustment for multiple comparisons
☐	☒	A full description of the statistical parameters including central tendency (e.g. means) or other basic estimates (e.g. regression coefficient) AND variation (e.g. standard deviation) or associated estimates of uncertainty (e.g. confidence intervals)
☐	☒	For null hypothesis testing, the test statistic (e.g. F, t, r) with confidence intervals, effect sizes, degrees of freedom and P value noted<br>*Give P values as exact values whenever suitable.*
☒	☐	For Bayesian analysis, information on the choice of priors and Markov chain Monte Carlo settings
☒	☐	For hierarchical and complex designs, identification of the appropriate level for tests and full reporting of outcomes
☐	☒	Estimates of effect sizes (e.g. Cohen's d, Pearson's r), indicating how they were calculated

Our web collection on statistics for biologists contains articles on many of the points above.

Software and code

Policy information about availability of computer code

Data collection	Zeiss LSM 880 Confocal Laser Scanning Microscope (Zeiss)<br>Zeiss AXIO Microscope (Zeiss)<br>SpectraMax® i3x Multi-Mode Microplate Reader (Molecular Devices)<br>ChemiDoc Touch Imaging System (BioRad)
Data analysis	Fiji ImageJ Software (Version 2.1.0)<br>Graphpad Prism version 9<br>Image Lab 6.0.1<br>ImageJ 1.53k<br>Bio-Rad CFX manager 3.1<br>SoftMax Pro 6 (6.4.2)<br>R (version 3.5.2)

For manuscripts utilizing custom algorithms or software that are central to the research but not yet described in published literature, software must be made available to editors and reviewers. We strongly encourage code deposition in a community repository (e.g. GitHub). See the Nature Portfolio guidelines for submitting code & software for further information.

Data

Policy information about availability of data

All manuscripts must include a data availability statement. This statement should provide the following information, where applicable:

- Accession codes, unique identifiers, or web links for publicly available datasets
- A description of any restrictions on data availability
- For clinical datasets or third party data, please ensure that the statement adheres to our policy

The data supporting the findings of this study are available within the paper and supplemental information files. All material, reagents, and experimental data are available from the corresponding authors upon request. No custom code were used in this study.

Research involving human participants, their data, or biological material

Policy information about studies with human participants or human data. See also policy information about sex, gender (identity/presentation), and sexual orientation and race, ethnicity and racism.

Reporting on sex and gender	To measure HSP70 and midkine level in serum and synovial fluids, a total of 22 non-OA and 165 knee symptomatic knee OA patients from the New York biomarker cohort were included in this study according to the American college of rheumatology (ACR) criteria, the demographic information has been previously described in "Attur, M. et al. Plasma levels of interleukin-1 receptor antagonist (IL1Ra) predict radiographic progression of symptomatic knee osteoarthritis. Osteoarthritis Cartilage 23, 1915-1924, doi:10.1016/j.joca.2015.08.006 (2015)."
Reporting on race, ethnicity, or other socially relevant groupings	Subjects are recruited without regard to race, ethnicity, and socioecomonic status.
Population characteristics	Human OA cartilage samples were harvested from patients receiving total knee joint replacement surgery for OA at New York University Langone Orthopaedic Hospital. Non-arthritic femoral condyle cartilage specimens were obtained from fresh osteochondral allografts discarded following donor plug harvesting during surgical osteochondral allograft implantation.
Recruitment	Human cartilage were collected from de-identified donors following informed consent.
Ethics oversight	Human subjects research was performed according to the Institutional Review Boards at New York University Medical Center (IRB Study Number i11-01488, i9018, and i05-131).

Note that full information on the approval of the study protocol must also be provided in the manuscript.

Field-specific reporting

Please select the one below that is the best fit for your research. If you are not sure, read the appropriate sections before making your selection.

☒ Life sciences ☐ Behavioural & social sciences ☐ Ecological, evolutionary & environmental sciences

For a reference copy of the document with all sections, see nature.com/documents/nr-reporting-summary-flat.pdf

Life sciences study design

All studies must disclose on these points even when the disclosure is negative.

Sample size	Sample sizes were not predetermined and were indicated in the figure legends. The sample size was decided based on effect sizes observed in preliminary experiments, prior experiments performed in our labs, or published findings.
Data exclusions	No data was excluded.
Replication	The number of biological replicates is as described in the figure legends. All attempts at replication were successful.
Randomization	For in vivo studies, age-matched mice were randomly assigned to treatment groups. For in vitro studies, cell culture experiments were pooled before splitting into individual identical wells followed by being randomly assigned to each experimental group.
Blinding	Experiments were performed blinded when possible. All the behavioral tests and histology evaluation were conducted in a blinded manner. For microscopy data collection and analysis, the field of view were chosen on a random basis, and were often performed by independent investigator blinded to group information, preventing biased selection of field with desired phenotype.

Reporting for specific materials, systems and methods

We require information from authors about some types of materials, experimental systems and methods used in many studies. Here, indicate whether each material, system or method listed is relevant to your study. If you are not sure if a list item applies to your research, read the appropriate section before selecting a response.

Materials & experimental systems

n/a	Involved in the study
☐	☒ Antibodies
☐	☒ Eukaryotic cell lines
☒	☐ Palaeontology and archaeology
☐	☒ Animals and other organisms
☒	☐ Clinical data
☒	☐ Dual use research of concern
☒	☐ Plants

Methods

n/a	Involved in the study
☒	☐ ChIP-seq
☒	☐ Flow cytometry
☒	☐ MRI-based neuroimaging

Antibodies

Antibodies used

Nav1.7 (Alomone Labs, Cat#ASC-008); TNFR2 (ProteinTech, Cat#19272-1-AP), GAPDH (ProteinTech, Cat#60004-1-Ig), NCX1 (Abcam, Cat#ab177952), Col2 (Invitrogen, Cat#MA5-12789), Mmp13 (Abcam, Cat#ab3208), aggrecan neoepitope (Novus Biologicals, Cat#NB100-74350), HSP70 (Invitrogen, Cat#MA3-009), Midkine (Abcam, Cat#ab170820)

Validation

All antibodies are from commercially available sources and have been validated by the manufacturers with supporting data and publications by other researchers. See below for a summary:

Nav1.7 (Alomone Labs, Cat#ASC-008)
Reactivity: Human, Mouse, Rat Applications: ICC, IF, IHC, WB

TNFR2 (ProteinTech, Cat#19272-1-AP)
Reactivity: Human, Mouse Applications: WB, IP, IF, FC, ELISA

GAPDH (ProteinTech, Cat#60004-1-Ig)
Reactivity: Human, Mouse, Rat, Yeast, Plant, Zebrafish Applications: WB, IP, IHC, IF, FC, CoIP, ChIP, Cell treatment, ELISA

NCX1 (Abcam, Cat#ab177952)
Reactivity: Human, Mouse, Rat Applications: WB

Col2 (Invitrogen, Cat#MA5-12789)
Reactivity: Bovine, Chicken, Human, Mouse, Rat Applications: WB, IHC, IF, Flow

Mmp13 (Abcam, Cat#ab3208)
Reactivity: Human, Mouse, Rat Applications: IHC, IF

aggrecan neoepitope (Novus Biologicals, Cat#NB100-74350)
Reactivity: Human, Mouse, Rat, Porcine, Bovine, Canine Applications: IHC, IF, Flow

HSP70 (Invitrogen, Cat#MA3-009)
Reactivity: Human, Mouse Applications: WB, IHC, Flow, Neutralization

Midkine (Abcam, Cat#ab170820)
Reactivity: Human, Mouse Applicaions: IHC, WB
It is reported to neutralize midkine in the following publication (Xiaofan Guo, Yuan Pan, Min Xiong, Shilpa Sanapala, Corina Anastasaki, Olivia Cobb, Sonika Dahiya & David H. Gutmann. Midkine activation of CD8+ T cells establishes a neuron–immune–cancer axis responsible for low-grade glioma growth. Nature Communications volume 11, Article number: 2177, 2020.)

Eukaryotic cell lines

Policy information about cell lines and Sex and Gender in Research

Cell line source(s)

C28I2 cell was purchased from Sigma (Cat#SCC043). Primary mouse and human chondrocytes were isolated from mouse or human cartilage.

Authentication

C28I2 is authenticated by Sigma.

Mycoplasma contamination

All cell used were mycoplasma-free.

Commonly misidentified lines
(See ICLAC register)

No commonly misidentified cell lines were used.

Animals and other research organisms

Policy information about studies involving animals; ARRIVE guidelines recommended for reporting animal research, and Sex and Gender in Research

Laboratory animals	C57BL/6 and Agc1-CreERT2 mice were obtained from The Jackson Laboratory (Bar Harbor, ME, USA). Nav1.8Cre;Nav1.7flox/flox mice were generously provided by Dr. John Wood at University College London, and mated with transgenic mice expressing Agc1-CreERT2 to obtain inducible Nav1.7 knockout mice in chondrocytes and both chondrocyte and DRGs. All animals were housed on a 12-hour light-dark cycle with ad libitum access to food and water in a specific pathogen-free environment. Animals were maintained on a C57BL/6J background, and age matched males typically at 12-weeks-of-age were used, if not otherwise specified in the figure legends.
Wild animals	This study did not involve wild animals.
Reporting on sex	Both female and male mice were used in the study.
Field-collected samples	This study did not involve samples collected from the field.
Ethics oversight	All animal studies were performed in accordance with institutional guidelines and approved by the Institutional Animal Care and Use Committee of New York University Grossman School of Medicine.

Note that full information on the approval of the study protocol must also be provided in the manuscript.
